# Mechanisms of Rice Endophytic Bradyrhizobial Cell Differentiation and Its Role in Nitrogen Fixation

**DOI:** 10.1264/jsme2.ME20049

**Published:** 2020-07-29

**Authors:** Teerana Greetatorn, Shun Hashimoto, Taro Maeda, Mitsutaka Fukudome, Pongdet Piromyou, Kamonluck Teamtisong, Panlada Tittabutr, Nantakorn Boonkerd, Masayoshi Kawaguchi, Toshiki Uchiumi, Neung Teaumroong

**Affiliations:** 1 School of Biotechnology, Institute of Agricultural Technology, Suranaree University of Technology, Nakhon Ratchasima 30000, Thailand; 2 The Center for Scientific and Technological Equipment, Suranaree University of Technology, Nakhon Ratchasima 30000, Thailand; 3 Graduate School of Science and Engineering, Kagoshima University, 890–0065 Kagoshima, Japan; 4 National Institute for Basic Biology, Nishigonaka 38, Myodaiji, Okazaki 444–8585 Aichi, Japan

**Keywords:** rice, endophyte, *Bradyrhizobium*, cell differentiation, nitrogen fixation

## Abstract

*Bradyrhizobium* sp. strain SUTN9-2 is a symbiotic and endophytic diazotrophic bacterium found in legume and rice plants and has the potential to promote growth. The present results revealed that SUTN9-2 underwent cell enlargement, increased its DNA content, and efficiently performed nitrogen fixation in response to rice extract. Some factors in rice extract induced the expression of cell cycle and nitrogen fixation genes. According to differentially expressed genes (DEGs) from the transcriptomic analysis, SUTN9-2 was affected by rice extract and the deletion of the *bclA* gene. The up-regulated DEGs encoding a class of oxidoreductases, which act with oxygen atoms and may have a role in controlling oxygen at an appropriate level for nitrogenase activity, followed by GroESL chaperonins are required for the function of nitrogenase. These results indicate that following its exposure to rice extract, nitrogen fixation by SUTN9-2 is induced by the collective effects of GroESL and oxidoreductases. The expression of the sensitivity to antimicrobial peptides transporter (*sapDF*) was also up-regulated, resulting in cell differentiation, even when *bclA* (*sapDF*) was mutated. This result implies similarities in the production of defensin-like antimicrobial peptides (DEFs) by rice and nodule-specific cysteine-rich (NCR) peptides in legume plants, which affect bacterial cell differentiation.

*Bradyrhizobium* spp. from symbiotic and endophytic relationships with legumes and non-legumes, such as rice, namely, *Oryza breviligulata* (Chaintreuil *et al.*, 2000) and *Oryza sativa* L. ssp. *indica* (Teamtisong *et al.*, 2014) and *japonica* (Piromyou *et al.*, 2015a, 2015b). *Bradyrhizobium* sp. strain SUTN9-2 is capable of forming symbiotic and endophytic relationships with legume and rice plants (Piromyou *et al.*, 2017; Greetatorn *et al.*, 2019). Biological nitrogen fixation (BNF) by endophytic bradyrhizobia in rice may be caused by the activity of nitrogenase enzyme, encoded by the gene *nifH* (the nitrogenase structural component) (Teamtisong *et al.*, 2014; Okubo *et al.*, 2016). Another gene, *nifV*, is involved in the biosynthesis of homocitrate synthase, which activates the nitrogenase Fe protein in free-living diazotrophs (Howard and Rees, 1994) and *Bradyrhizobium* sp. (Pagan *et al.*, 1975; Okubo *et al.*, 2016). The *nifV* gene was also found in *Bradyrhizobium* sp. SUTN9-2 (Noisangiam *et al.*, 2012; Hashimoto *et al.*, 2019). This gene is mostly absent in *Rhizobium* sp. that efficiently perform nitrogen fixation only in symbiosis with legumes (Hakoyama *et al.*, 2009). This finding indicates its potential as a candidate for use as a biofertilizer or bioinoculant. However, there is currently no information on the rice endophytic molecular mechanisms that play important roles in plant colonization and growth promotion.

Elongated SUTN9-2 cells were recently observed in rice tissues at 7 days post-inoculation (dpi) by scanning electron microscopy (SEM). The elongation of these cells occurred between 3 (≅1–2 micrometer [μm]) and 7 (≅3‍ ‍μm) dpi (Piromyou *et al.*, 2017). Based on this finding, we hypothesize that interactions between rice plants and bacterial factors may contribute to cell size enlargements and increases in nitrogen fixation efficiency by SUTN9-2 in rice plants, similar to bacteroid differentiation in legume plants. The responses of endophytic *Burkholderia kururiensis* M130 to rice macerate have been investigated using a transcriptomic analysis. The findings obtained revealed 27.7% of differentially expressed genes (DEGs) of its open reading frames in the presence of rice macerate. These genes were involved in membrane transporter and secretion systems, motility, chemotaxis, and adhesion, indicating the importance of the exchange of molecules for bacterial endophytic growth and adaptation to rice plants (Coutinho *et al.*, 2015).

The terminal bacteroid differentiation (TBD) of *Rhizobium* and *Bradyrhizobium* spp. has been extensively examined, particularly in an Inverted Repeat-Lacking Clade (IRLC) producing elongated polyploid bacterial cells that switch their cell cycle towards endoreduplication (Mergaert *et al.*, 2006; Kondorosi *et al.*, 2013; Alunni and Gourion, 2016). TBD is assessed by host plant factors, including defensin-like antimicrobial peptides (DEFs) consisting of nodule-specific cysteine-rich (NCR) peptides, which are produced in large families of IRLC and Delbergioid legume clades, together with the BacA transporter protein in microbes (Mergaert *et al.*, 2006; Alunni and Gourion, 2016). NCR peptides and the BacA transporter protein have been shown to mediate the polyploidy of *Sinorhizobium meliloti* in *Medicago* nodules by altering the processes involved in sequential changes in the expression of cell cycle genes and cell size enlargements (De Nisco *et al.*, 2014; Penterman *et al.*, 2014). The BacA-like transporter of *Bradyrhizobium* sp. strain ORS285 also provides protection against the antimicrobial activity of NCR peptides in *Aeschynomene* spp. nodules. The BacA-like transporter has been identified in ORS285, carrying three genes (BRAO285v1_1320006, BRAO285v1_250005, and BRAO285v1_950010). These genes are characterized by the presence of the transmembrane domain pfam06472 (ABC_membrane_2) or pfam05992 (SbmA_BacA). However, only BRAO285v1_1320006 plays a key role in the symbiotic phenotype in host plants, providing protection against the antimicrobial activity of NCR peptides in *Aeschynomene* spp. nodules. The mutant produced small nodules, undifferentiated bacteroids, reduced nitrogen fixation activity in *Aeschynomene indica* and *Aeschynomene afraspera*, and some dead cells were observed in *A. indica* nodules. The other two genes formed normal symbiosis nodules, differentiated bacteroids, and nitrogen fixation, indicating that these genes are not important for ORS285 in *Aeschynomene* spp. symbiosis (Guefrachi *et al.*, 2015). These findings suggested that BacA-like proteins in ORS285 were encoded by BRAO285v1_1320006. However, *Bradyrhizobium* BacA-like (BclA), named according to BacA or the *Escherichia coli* homolog SbmA, differs from *Bradyrhizobium* proteins due to the presence of a C-terminal cytosolic ATPase domain typical for canonical ABC transporters (Guefrachi *et al.*, 2015). Furthermore, a correlation was observed between cell differentiation and nitrogen fixation activity in alfalfa (*Medicago sativa*) nodules (Vasse *et al.*, 1990). The small bacteroid size (1–2.5‍ ‍μm) with a low nucleic acid content also exhibited weak acetylene reduction activity. On the other hand, the enlarged bacteroid size (5–7‍ ‍μm) had a high nucleic acid content and very strong acetylene reduction activity (Paau and Cowles, 1978). This finding indicated a correlation between cell size and nitrogen fixation activity. Thus, the effects of DEFs from rice plants and the BclA of SUTN9-2 on cell size enlargement and nitrogen fixation efficiency, occurring during the interaction between SUTN9-2 and rice extract, were analyzed. The results obtained provide a better understanding of the mechanisms and factors involved in cell differentiation and nitrogen fixation in this model.

## Materials and Methods

### Plants and the bacterial strain

The rice plants *O. sativa* L. ssp. *indica* cv. Pathum Thani 1 and *O. sativa* L. ssp. *japonica* cv. Nipponbare were used in the present study. *Bradyrhizobium* sp. strain SUTN9-2 WT (LAXE00000000) was isolated from the root and stem nodules of *A. americana*, grown in rice field areas in Thailand (Noisangiam *et al.*, 2012). SUTN9-2 DsRed-tagged (Piromyou *et al.*, 2015b), Δ*nifV*, and Δ*bclA* were also used in the present study. SUTN9-2 WT and mutants were cultured at 30±2°C in yeast extract-mannitol (YEM) broth medium (Somasegaran and Hoben, 2012) for further analyses. The medium was supplemented with 200‍ ‍μg mL^–1^ of streptomycin and spectinomycin for SUTN9-2 DsRed-tagged, 20‍ ‍μg mL^–1^ of cefotaxime for Δ*nifV*, and 200‍ ‍μg mL^–1^ of streptomycin for Δ*bclA*.

### Rice growth and rice extract preparation

Rice seeds (*O. sativa* L. ssp. *indica* cv. Pathum Thani 1 and *O. sativa* L. ssp. *japonica* cv. Nipponbare) were dehulled and surface sterilized with 70% ethanol for 3‍ ‍min, twice with 10% hydrogen peroxide for 10‍ ‍min, with 3% sodium hypochlorite for 1 h, and then washed 3 times with sterilized water (Greetatorn *et al.*, 2019). To obtain a small emerging root, surface-disinfected rice seeds were germinated on 0.85% agar with YEM medium at 37°C for 1 day in the dark. Three germinated seeds (no contamination on YEM) were transplanted into glass test tubes (22×200‍ ‍mm) containing a sterilized aluminum net and N-free rice nutrient solution ([mmol L^–1^]: NaH_2_PO_4_·2H_2_O, 0.6; K_2_SO_4_, 0.3; CaCl_2_·2H_2_O, 0.3; MgCl_2_·6H_2_O, 0.6; EDTA-Fe, 0.045; H_3_BO_3_, 0.05; MnSO_4_·5H_2_O, 0.009; CuSO_4_·5H_2_O, 0.0003; ZnSO_4_·7H_2_O, 0.0007; and Na_2_MoO_4_·2H_2_O, 0.0001) and pH adjusted to 6.8 (Mae and Ohira, 1981). Rice plants were grown for 14 days under controlled environmental conditions at 28±2°C and 70% relative humidity on a 16:8-h day:night cycle (full light, 639 microeinsteins [μE] m^–2^ S^–1^).

Whole rice samples (root+shoot; 0.5‍ ‍g plant^–1^) were harvested at 14 dpi, sterilized and macerated separately with a sterilized mortar and pestle in sterilized buffered nodulation medium B (BNM-B) minimal medium, and kept at 4°C for no more than 3 days for further analyses. BNM-B is a synthetic plant growth medium (Ehrhardt *et al.*, 1992) supplemented with succinate, glutamate, and a cocktail of vitamins (Renier *et al.*, 2011). The rice extract was used by supplementing BNM-B medium (20–25‍ ‍g of rice extract 400‍ ‍mL^–1^ in BNM-B). The homogenate was passed through three layers comprising a miracloth (22–25‍ ‍μm), membrane filter (8‍ ‍μm), and syringe filter (0.2‍ ‍μm) to discard plant debris and the rice extract obtained was further incubated with 10^8^ SUTN9-2 cells in a 10-mL test tube with a tight cap and stable stage.

### Construction of SUTN9-2 *bclA* and *nifV* mutants

The *bclA* deletion mutant (SUTN9-2Δ*bclA*) was constructed and standard molecular techniques were used in the present study. The 700-bp upstream and downstream fragments of the *bclA* gene were amplified by PCR and the primers used are listed in Table S1. The two regions were merged by overlap extension PCR and then digested by *EcoR*I/*Xba*I and cloned into the plasmid pNPTS129. This plasmid cannot replicate in *Bradyrhizobium* strains and carries *sacB* and the kanamycin resistance gene, which confer sensitivity to sucrose, thereby inducing bacterial death (Tsai and Alley, 2000). The spectinomycin and streptomycin cartridge from pHP45Ω was digested by *BamH*I and introduced into the upstream and downstream regions previously cloned in the pNPTS129 plasmid. The plasmid was then transferred into SUTN9-2 by triparental conjugation with the helper plasmid pRK2013 (Tamura *et al.*, 2011). Single recombinant clones were obtained by antibiotic selection, followed by double recombinant clones by growth on sucrose with spectinomycin and streptomycin, but not kanamycin. Candidate clones were checked for the loss of kanamycin resistance from the pNPTS129 plasmid, and the deletion of the *bclA* gene was verified by PCR. The mutation of the *nifV* gene was constructed as described by Hashimoto *et al.* (2019).

### Confocal laser scanning microscopy

SUTN9-2 DsRed-tagged was treated with BNM-B and BNM-B supplemented with rice extract (*O. sativa* L. ssp. *Indica* cv. Pathum Thani) for 7, 14, 21, and 28 days. SUTN9-2 WT, Δ*nifV*, and Δ*bclA* were treated with rice extract BNM-B and BNM-B supplemented with rice extract from *indica* and *japonica* for 7, 14, 21, and 28 days, and were then collected and stained with 30‍ ‍μg mL^–1^ DAPI and 15‍ ‍μg mL^–1^ FM4-64. Cell size, DNA content, and the cell membrane were observed in all treated cells under a confocal laser scanning microscope (Nikon Model Ni-E; *Nikon* Instech). The mean DNA content area was calculated from each cell (μm^2^) using an ImageJ analysis (Collins, 2007), and the average enlarged cell size in each treatment was calculated from 20 cells in each replication for 3 replications.

### Flow cytometry analysis

SUTN9-2 WT and DsRed-tagged cells were treated with BNM-B and BNM-B supplemented with rice extract (*O. sativa* L. ssp. *Indica* cv. Pathum Thani 1) for 7, 14, and 28 days. SUTN9-2 WT-treated cells were fixed at 4°C overnight in 70% ethanol, the fixed sample was digested with RNase (DNase free), and then stained with 50‍ ‍μg mL^–1^ propidium iodide (PI) to analyze the DNA content (Deitch *et al.*, 1982). The size of SUTN9-2 DsRed-tagged cells treated as described above was assessed. The analysis of cell size and DNA content was conducted using a flow cytometer (BD FACSCalibur, BD Bioscience) with Cyflowgic software.

### Acetylene reduction assay (ARA)

The effects of rice extract on the nitrogen fixation efficiency of SUTN9-2 WT (DsRed-tagged), Δ*nifV*, and Δ*bclA* cells treated with rice extract were investigated using ARA (Chaintreuil *et al.*, 2000). The reactions were performed in a 10-mL test tube containing 2‍ ‍mL each of BNM-B medium and BNM-B medium supplemented with rice extract (*O. sativa* L. ssp. *indica* cv. Pathum Thani 1 and *japonica* cv. Nipponbare) as described previously. The reaction was incubated at 28±2°C for 7, 14, 21, and 28 days. Acetylene was injected to reach a final concentration of 10% (v/v) of the gas phase in the headspace (Somasegaran and Hoben, 2012). After the incubation, gas from the vessel was injected into a gas chromatograph (6′×1/8″ S.S. Hayesep T column; Valco Instruments). Ethylene gas production was analyzed, as described by Renier *et al.* (2011). Total concentrations in the cell suspension were evaluated using a plate count of colony-forming units (CFU) on YEM medium. The medium was supplemented with 200‍ ‍μg mL^–1^ of streptomycin and spectinomycin for SUTN9-2 DsRed-tagged cells, 20‍ ‍μg mL^–1^ of cefotaxime for Δ*nifV*, and 200‍ ‍μg mL^–1^ of streptomycin for Δ*bclA*.

### Rice cultivation and growth promotion

The rice seeds of *O. sativa* L. ssp. *indica* cv. Pathum Thani 1 were surface sterilized and germinated as described above. Germinated seeds were soaked overnight in YEM broth containing SUTN9-2 WT, Δ*nifV*, and Δ*bclA* (10^8^ CFU mL^–1^), and three germinated seeds were then transplanted into glass test tubes (22×200‍ ‍mm) containing a sterilized aluminum net with N-free rice nutrient solution and N-free supplemented with 1‍ ‍mmol L^–1^ ammonium nitrate under controlled environmental conditions, as previously described. Rice plants were grown and harvested at 7, 14, 21, and 28 dpi, whole rice samples were then dried in an oven at 65°C for 72 h, and dry weights were measured.

### RNA preparation for qRT-PCR

RNA was isolated from three independent cultures of SUTN9-2 WT, Δ*bclA*, and Δ*nifV* incubated in BNM-B medium or BNM-B medium supplemented with rice extract (*indica*). The cultures were incubated in a 10-mL test tube with a tight cap at 28±2°C for 21 days. RNA was isolated from 10^8^ cells using Plant RNA extraction kits (Qiagen). RNAs from rice plant samples (shoot+root) were harvested at 21 dpi, sterilized, and macerated (Greetatorn *et al.*, 2019). Total RNA extraction was performed according to the manufacturer’s procedure. RNAs were treated with DNaseI (Qiagen) at 28±2°C for 30‍ ‍min to prevent the contamination of genomic DNA. The purity of RNA was assessed by PCR on total RNA (250‍ ‍ng) with GoTaq polymerase (Promega) using *dnaK*_SUNT9-2 primers and *EF-1∝*_rice plants (Table S1). The quality and concentration of RNA were assessed by Nanodrop (Thermo Scientific) and agarose gel electrophoresis.

### qRT-PCR and analysis

Transcription levels were measured by qRT–PCR using Applied Biosystem, QuantStudio Design (Waltham). Primers for the amplification of genes involved in the cell cycle (*dnaK*, *gcrA*, *ctrA*, *dnaA*, and *GcrM*), nitrogen fixation (*nifH* and *nifV*), and rice hemoglobin (*EF-1∝*, *hb1*, and *hb5*) are listed in Table S1. PCR amplification was performed under the following cycling conditions: an initial denaturation step at 95°C for two min, 35 cycles at 95°C for 2‍ ‍min and at the annealing temperature of all genes (50°C) for 30‍ ‍s, followed by a final 5-min extension at 72°C. Relative gene expression was analyzed by the comparative Ct method (-ΔΔCT) normalized to the endogenous housekeeping gene, *dnaK* for bacterial SUTN9-2 and *EF-1∝* for rice plants. Three biological replicates were pooled and analyzed.

### RNA preparation for the RNAseq analysis

RNA was isolated from three independent cultures of SUTN9-2 WT and Δ*bclA* incubated in BNM-B medium and BNM-B medium supplemented with rice extract (*indica*). Cultures were incubated in a 50-mL test tube with a tight cap at 28±2°C for 21 days. RNA was isolated from 3×10^8^ cells using Plant RNA extraction kits (Qiagen). Total RNA was extracted according to the manufacturer’s procedure. The purity of RNA was assessed as described above.

### RNA sequencing and analysis

To identify the bacterial genes that respond to rice extract, we used a comparative RNAseq analysis of SUTN9-2. RNA-Seq libraries were constructed from the RNA sample incubated with or without rice extract (20–25‍ ‍g of rice extract 400‍ ‍mL^–1^ in BNM-B) for SUTN9-2 WT and the Δ*bclA* mutant. Three biological replicates were prepared for each treatment. RNA samples were extracted following the same protocol for qRT-PCR. Eukaryotic rRNA from the samples was removed using the Ribo-Zero Magnetic Kit (Illumina) and stranded RNA-Seq libraries were constructed with the TruSeq Stranded mRNA kit (Illumina). Paired-end sequencing (150 bp) of the libraries was performed by the NovaSeq platform (Illumina). The libraries were constructed and sequenced at Novogene.

In the gene expression analysis, we adapted the mapping of the transcriptomic data obtained to the previously reported SUTN9-2 genome. A total of 865M reads (R1+R2) were obtained from the 12 libraries (Table S3). Adapter sequences and low-quality sequences were removed from raw single-end reads using fastp (v0.20.0) (fastp: an ultra-fast all-in-one FASTQ preprocessor [Chen *et al.*, 2018]). The trimmed paired-end reads were mapped to the SUTN9-2 genome (INSDC ID ASM312264v1) and calculated the TPM-normalized mapped read numbers by RSEM (v1.3.1) (RSEM: accurate transcript quantification from RNA-Seq data with or without a reference genome, [Li and Dewey, 2011]) with the --bowtie2 option (bowtie2 version 2.3.5.1) (Fast gapped-read alignment with Bowtie 2, [Langmead and Salzberg, 2012]). The mapped reads were counted based on the gene models of SUTN9-2 (LAXE01000000) predicted by Piromyou *et al.* (2015b). Differentially expressed genes (DEGs) were detected based on the false discovery rate (FDR) (<0.1) and log fold change (logFC) (>0.1 or <–0.1) from the RSEM analysis. In the enrichment analysis, the public gene models from LAXE01000000 were annotated with interproscan (v5.36-75) with --goterms option (for gene ontology annotation) and with KofamKOALA (downloaded on 13^th^ Aug. 2019) (KofamKOALA: KEGG Ortholog assignment based on the profile HMM and adaptive score threshold [Aramaki *et al.*, 2020] for KEGG Orthologs annotation). Enrichment analyses were performed by goseq (v1.38.0) on R (v3.6.1) (gene ontology analysis for RNA-seq: accounting for selection bias, [Young *et al.*, 2010]).

### qRT-PCR validation

To validate the results of the transcriptome analysis, significant DEGs were selected to compare their expression in SUTN9-2 WT in BNM-B with rice extract and SUTN9-2 WT in BNM-B alone, including biphenyl-2,3-diol 1,2-dioxygenase (*hpaD*), 3-(3-hydroxyphenyl) propanoate hydroxylase (*mhpA*), chaperonin GroEL (*groEL*), chaperonin GroES (*groES*), ABC transporter ATP-binding protein (*sapDF*), ABC transporter substrate-binding protein (*sapA*), and RND family efflux transporter (*cusF*). Primers for the amplification of these genes are listed in Table S1. RNA preparation, qRT-PCR, and analyses were performed as described above.

### SbmA_BacA domain proteins and phylogenetic analysis

The identified protein sequences of the SbmA_BacA domain, including the BacA, *Bradyrhizobium* homologous, ExsX, and *Mycobacterium* BacA clades, were obtained from Guefrachi *et al.* (2015). The BacA-related protein sequences of SUTN9-2 (BclA; PWE81210.1, SapA; PWE77331.1, and SapDF; PWE82048.1) were obtained from the National Center for Biotechnology Information (NCBI) database. Protein sequences were aligned using the ClustalW program. The phylogenetic tree was constructed using the neighbor-joining method with confidence levels for 500 replicates using the MEGAX package (Saitou and Nei, 1987; Kumar *et al.*, 2008).

### Statistical analysis

The statistical analysis of data sets was performed with SPSS software (SPSS 16.0 for Windows; SPSS) on data from three independent samples (each with three technical replicates). Experimental data were statistically analyzed according to Steel and Torrie (1980), and means were compared by Duncan’s multiple range test (*P*≤0.05) (Duncan, 1955).

## Results

### Cell size enlargement by SUTN9-2 in rice (*indica*) plants

To confirm whether SUTN9-2 cell size enlargement occurs within rice plants, the red fluorescent-tagged SUTN9-2 strain (DsRed) was extracted from rice plant tissues (*indica*) at 21 and 28 dpi and visualized using confocal laser scanning microscopy. The cells of SUTN9-2 were longer, with average sizes of 5.6 and 5.8‍ ‍μm at 21 and 28 dpi, respectively, than free-living SUTN9-2 cells (2.8‍ ‍μm) (Fig. S1). These results revealed that SUTN9-2 cell size increased within rice plants. However, small numbers of SUTN9-2 cells were extracted from rice plant tissues. Therefore, rice extract was prepared and subsequent experiments were performed by incubating SUTN9-2 cells with the extract to investigate the influence of rice and its derived molecules on the differentiation of SUTN9-2 cells.

### SUTN9-2 increases its cell size and DNA content in response to the rice extract (*indica*) treatment

**Fig. 1.**
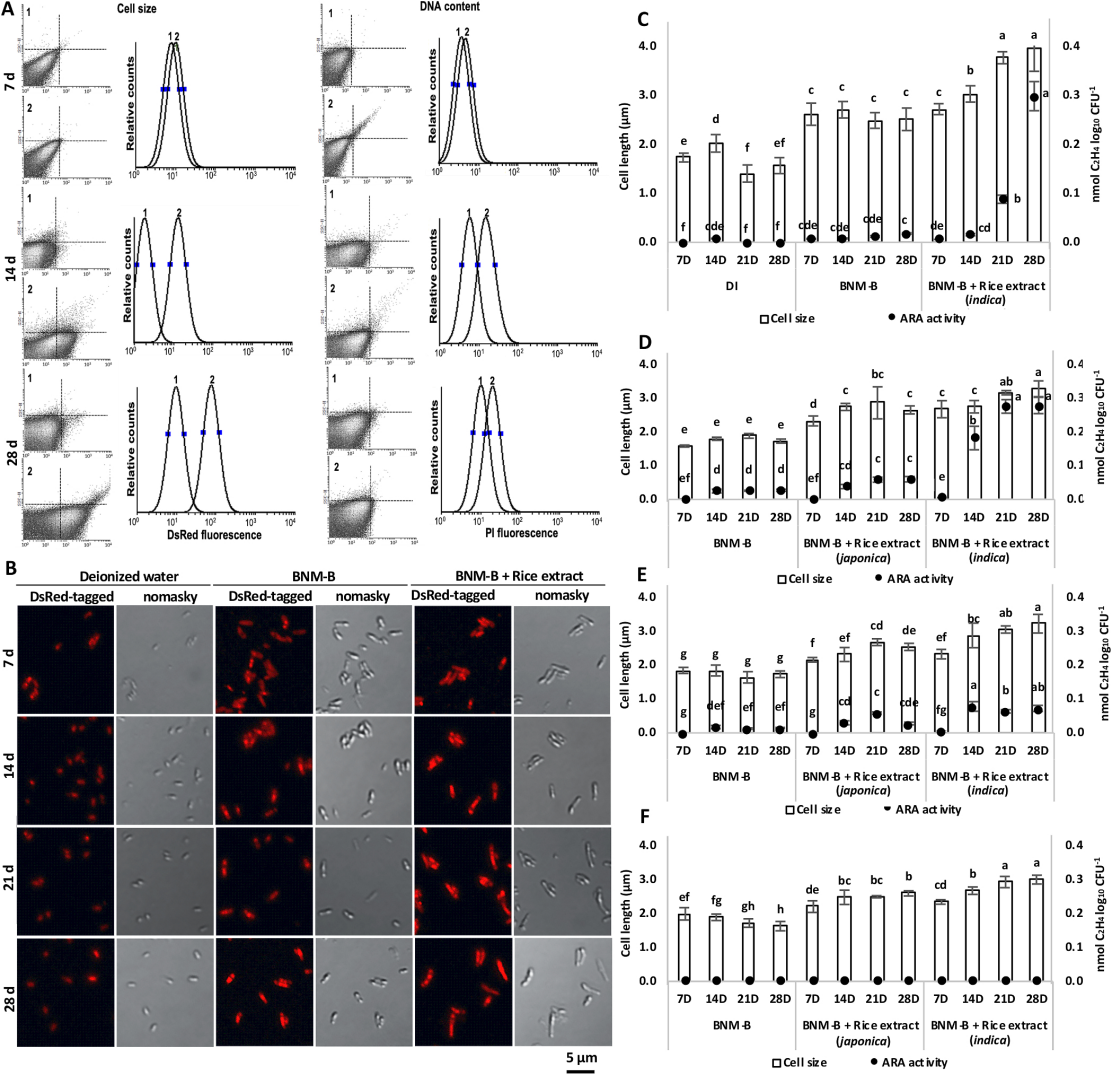
Cell size, DNA content, and nitrogenase activity of SUTN9-2 in response to rice extract (*indica*). Cell size, enlarged DsRed-tagged cells, and propidium iodide (PI)-stained DNA content were analyzed by flow cytometry (1 represents free growing, and 2 represents cells treated with rice extract) (A). Cell size and enlarged DsRed-tagged cells by confocal laser scanning microscope (B). Observation of cell size by a confocal laser scanning microscope and nitrogenase activity by the acetylene reduction assay of SUTN9-2 WT cells treated with DI, BNM-B, and BNM-B+rice extract (*indica*) (C), and SUTN9-2 WT (D), Δ*bclA* (E), and Δ*nifV* (F) with BNM-B, BNM-B+rice extract (*japonica*), and BNM-B+rice extract (*indica*) at 7, 14, 21, and 28 days. Significance at *P*≤0.05 is indicated by the mean±standard deviation (*n*=3).

Increases in cell size and DNA content in SUTN9-2 cells in response to the rice extract (*indica*) treatment were examined using a flow cytometer. The sizes of treated SUTN9-2 DsRed-tagged cells in BNM-B minimal medium and BNM-B supplemented with rice extract were analyzed. The forward scatter (FS) of a treated cell is related to its size. BNM-B with rice extract-treated cells had a higher FS than BNM-B-treated cells at 7, 14, and 28 days (Fig. 1A). Furthermore, the DNA content of treated SUTN9-2 WT cells was assessed by the fluorescent staining of nuclei using PI. The DNA content of BNM-B with rice extract-treated SUTN9-2 cells was higher than that in BNM-B-treated cells. The DNA content of BNM-B-treated cells was 1 to 2 genome complement (1C to 2C), similar to the ploidy level of free-growing cells (1C to 2C) (Czernic *et al.*, 2015), whereas BNM-B with rice extract-treated cells reached 2C to 7C ploidy levels, similar to the ploidy level of bacteroid cells (7C to 16C) (Czernic *et al.*, 2015; Guefrachi *et al.*, 2015) (Fig. 1A). These results confirmed that differentiated SUTN9-2 cells affected rice plants.

### SUTN9-2 cells increase their size and nitrogen fixation activity in response to rice extract (*indica*)

To investigate whether an enlarged cell size increases nitrogen fixation activity in response to rice extract, SUTN9-2 DsRed-tagged cells treated with deionized water (DI), BNM-B, and BNM-B with rice extract (*indica*) were analyzed. Cell size was larger in the BNM-B with rice extract group than in the BNM-B alone and DI groups. The average sizes of BNM-B with rice extract-treated cells at 7, 14, 21, and 28 days were 2.76, 3.18, 3.38, and 3.79‍ ‍μm, respectively (Fig. 1B), and average nitrogenase activities were 0.01, 0.02, 0.09, and 0.30 nmol C_2_H_4_ log_10_^–1^ CFU, respectively (Fig. 1C). The average sizes of BNM-B alone-treated cells at 7, 14, 21, and 28 days were with 2.52, 2.74, 2.42, and 2.64‍ ‍μm, respectively (Fig. 1B). Average nitrogenase activities in this group were 0.01 nmol C_2_H_4_ log_10_^–1^ CFU at 7, 14, and 21 days and 0.02 nmol C_2_H_4_ log_10_^–1^ CFU at 28 days (Fig. 1C). The average sizes of DI-treated cells at 7, 14, 21, and 28 days were 1.86, 2.04, 1.36, and 1.37‍ ‍μm, respectively (Fig. 1B), and these cells only exhibited nitrogenase activity of 0.01 nmol C_2_H_4_ log_10_^–1^ CFU at 14 days (Fig. 1C). These results demonstrated the influence of rice extract on the cell size and nitrogen fixation efficiency of SUTN9-2 cells. Time-dependent increases were observed in elongated cells in the BNM-B with rice extract group, but not in the BNM-B alone or DI group (Fig. 1B and C).

### SUTN9-2 WT and mutants show increases in cell size, DNA content, and nitrogen fixation activity in the presence of rice extract (*indica* and *japonica*)

To clarify the effects of rice species variations and *bclA* and *nifV* genes on cell differentiation and nitrogen fixation, SUTN9-2 WT, Δ*bclA*, and Δ*nifV* were treated with rice extract from 2 different species of rice (*indica* and *japonica*). Cell size, DNA content, and nitrogenase activity were increased in the BNM-B with rice extract (*indica*) group at 28 days with average cell sizes of 3.28, 3.22, and 3.03‍ ‍μm (Fig. 1D, E, and F), mean DNA content areas of 0.43, 0.33, and 0.32‍ ‍μm^2^ (Fig. S2 and S3), and nitrogenase activities of 0.28, 0.07, and 0.00 nmol C_2_H_4_ log_10_^–1^ CFU in SUTN9-2 WT, Δ*bclA*, and Δ*nifV*, respectively (Fig. 1D, E, and F). The BNM-B with rice extract (*japonica*) group showed average cell sizes of 2.66, 2.56, and 2.60‍ ‍μm (Fig. 1D, E, and F), mean DNA content areas of 0.32, 0.33, and 0.28‍ ‍μm^2^ (Fig. S2 and S3), and nitrogenase activities of 0.06, 0.03, and 0.00 nmol C_2_H_4_ log_10_^–1^ CFU for SUTN9-2 WT, Δ*bclA*, and Δ*nifV*, respectively (Fig. 1D, E, and F). Average cell sizes at 28 days in BNM-B-treated cells were 1.73, 1.73, and 1.63‍ ‍μm (Fig. 1D, E, and F), mean DNA content areas of 0.11, 0.15, and 0.15‍ ‍μm^2^ (Fig. S2 and S3), and nitrogenase activities of 0.05, 0.01, and 0.00, nmol C_2_H_4_ log_10_^–1^ CFU in SUTN9-2 WT, Δ*bclA*, and Δ*nifV*, respectively (Fig. 1D, E, and F). Similar results were obtained at 7, 14, and 21 days (Fig. 1D, E, and F). The effects of rice extract and Δ*bclA* and Δ*nifV* on cell sizes were observed under a transmission electron microscope (TEM). The cells of SUTN9-2 WT, Δ*bclA*, and Δ*nifV* elongated in response to the rice extract (*indica*) treatment (Fig. S4).

These results demonstrated that rice extract (*indica*) exerted stronger effects on cell elongation and nitrogen fixation activity than rice extract (*japonica*) in WT and mutant SUTN9-2 cells. Increases in cell size and nitrogenase activity were smaller in Δ*bclA* than in the WT strain. Furthermore, nitrogenase activity was not detected in Δ*nifV* and their cell size was smaller than those of WT and Δ*bclA* following the treatment with both rice extracts. These results also revealed the effects of *bclA* and *nifV* genes on cell size enlargement and nitrogen fixation activity in response to different rice varieties.

### Effects of WT and mutant SUTN9-2 on rice (*indica*) plant growth

SUTN9-2 has been reported to promote rice (*indica*) growth at the early seedling stage (Greetatorn *et al.*, 2019). Consistent results were obtained in the present study; SUTN9-2 WT, Δ*bclA*, and Δ*nifV* increased rice dry weight more than the non-inoculated control, particularly at 7 and 14 dpi, in N-free medium and ammonium nitrate-supplemented medium (Fig. S5). The significant difference observed at 7 and 14 dpi between rice plants inoculated with SUTN9-2 WT, Δ*bclA*, and Δ*nifV* and the non-inoculated control was not detected at 21 or 28 dpi (Fig. S5). However, rice dry weight did not show a significant difference between rice plants inoculated with SUTN9-2 WT, Δ*bclA*, and Δ*nifV* at 7, 14, 21, and 28 dpi (Fig. S5). These results clearly demonstrated that SUTN9-2 promoted rice plant growth at the early stage at 7 and 14 dpi under the presence or absence of supplementation with a nitrogen source. However, the effects of *bclA* and *nifV* genes on rice plant growth remain unclear.

### Expression of cell cycle genes in WT and mutant SUTN9-2 in response to rice extract (*indica*)

**Fig. 2.**
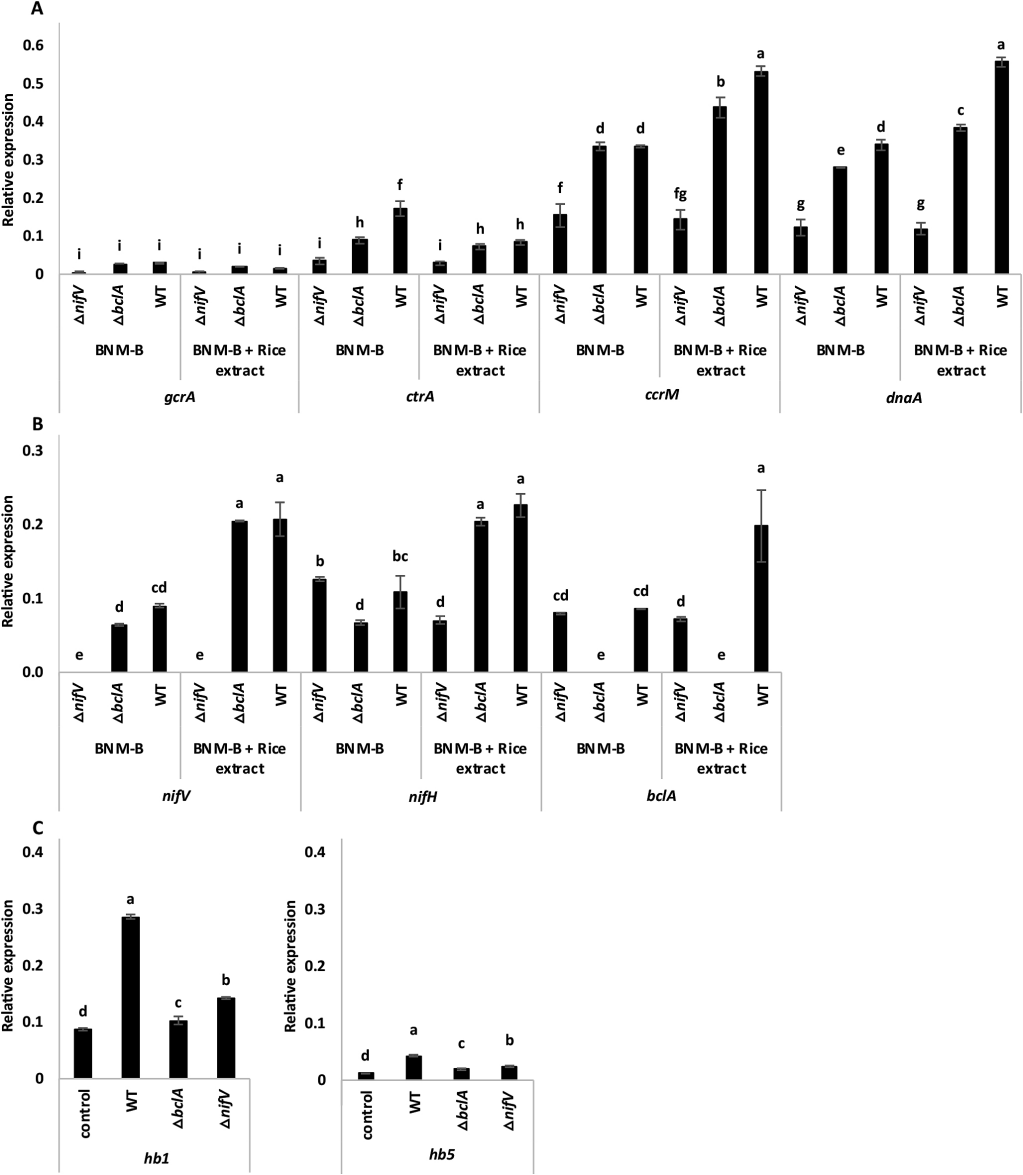
Relative expression of genes involved in the master cell cycle (A), nitrogen fixation and BclA transporter (B) of SUTN9-2 Δ*nifV*, Δ*bclA*, and WT in response to rice extract (*indica*) at 21 days. Relative expression of the rice hemoglobin gene (*hb1* and *hb5*) in response to SUTN9-2 Δ*nifV*, Δ*bclA*, and WT at 21 dpi (C). Significance at *P*≤0.05 was indicated by the mean standard deviation (*n*=3).

SUTN9-2 cell differentiation-related genes, including *gcrA*, *ctrA*, *ccrM*, and *dnaA*, were selected based on their role in the master cell cycle. Similar results were obtained for the expression of all genes between SUTN9-2 WT and Δ*bclA*. However, Δ*bclA* showed the down-regulated expression of the genes listed above. The expression levels of *gcrA* and *ctrA* were lower in the BNM-B with rice extract (*indica*) group than in the BNM-B alone group, with 0.01- and 0.09-fold differences in WT and 0.008- and 0.01-fold differences in Δ*bclA*, respectively (Fig. 2A). However, no significant difference was noted in *gcrA* expression between the BNM-B alone and BNM-B with rice extract groups. In contrast, the expression levels of *ccrM* and *dnaA* were higher in the BNM-B with rice extract group than in the BNM-B alone group, with 0.20- and 0.22-fold differences for WT and 0.10- and 0.10-fold differences for Δ*bclA*, respectively (Fig. 2A). In spite of this result, the expression of all genes in Δ*nifV* was several-fold lower in the BNM-B with rice extract group than in the BNM-B alone group (Fig. 2A). These results suggested that cell size enlargements may also be affected by the *nifV* gene, supporting the observation of cell enlargement under the microscope. They also indicated that the *bclA* gene was disrupted, whereas other *bclA*-associated genes were still active and required for cell enlargement, showing a similar pattern of gene expression to WT. Collectively, these results demonstrated that some factors from rice extract may affect master cell-cycle regulators.

### Nitrogen fixation and BclA transporter gene expression in WT and mutant SUTN9-2 in response to rice extract (*indica*)

To examine the effects of rice extract on BclA transporter and nitrogen fixation-related gene expression in SUTN9-2, their relative expression levels were measured in SUTN9-2 WT, Δ*bclA*, and Δ*nifV*. The expression levels of the *nifH*, *nifV*, and *bclA* genes were significantly increased in WT (0.12-, 0.12-, and 0.11-fold, respectively) and Δ*bclA* (*nifH*; 0.14-, and *nifV*; 0.14-fold) in the presence of rice extract (Fig. 2B). In contrast, the expression levels of these genes were significantly decreased in Δ*nifV* (*nifH*; 0.06-, *bclA*; 0.008-fold) in the presence of rice extract (Fig. 2B). However, the expression of the *bclA* and *nifV* genes was not detected in SUTN9-2 mutants lacking *bclA* and *nifV*, respectively (Fig. 2B). The results demonstrated that rice extract affected BclA transporter and nitrogen fixation-related gene expression, indicating the effect of Δ*nifV* on *nifH* and *bclA* gene expression.

### Expression of rice (*indica*) hemoglobin genes in response to WT and mutant SUTN9-2

To assess the relationship between hemoglobin gene expression and nitrogen fixation efficiency by SUTN9-2 in rice plants, *hb1* and *hb5* gene expression levels were assessed in rice plants inoculated with SUTN9-2 WT, Δ*bclA*, and Δ*nifV*. The results obtained showed that *hb1* and *hb5* expression was more strongly induced in rice inoculated with SUTN9-2 WT (0.2- and 0.03-fold), Δ*bclA* (0.02- and 0.007-fold), and Δ*nifV* (0.06- and 0.01-fold) than with the non-inoculated control (Fig. 2C). The expression of these genes was suppressed in Δ*bclA*- and Δ*nifV*-inoculated rice, with the expression level of the *hb5* gene being lower than that of the *hb1* gene (Fig. 2C). These results indicated that the activity of hemoglobin in rice plants may be induced in response to the SUTN9-2 inoculation and may function as an oxygen scavenger or oxygen stock to facilitate nitrogen fixation in the endophytic state of SUTN9-2.

### SUTN9-2 transcriptome in response to rice extract (*indica*)

**Fig. 3.**
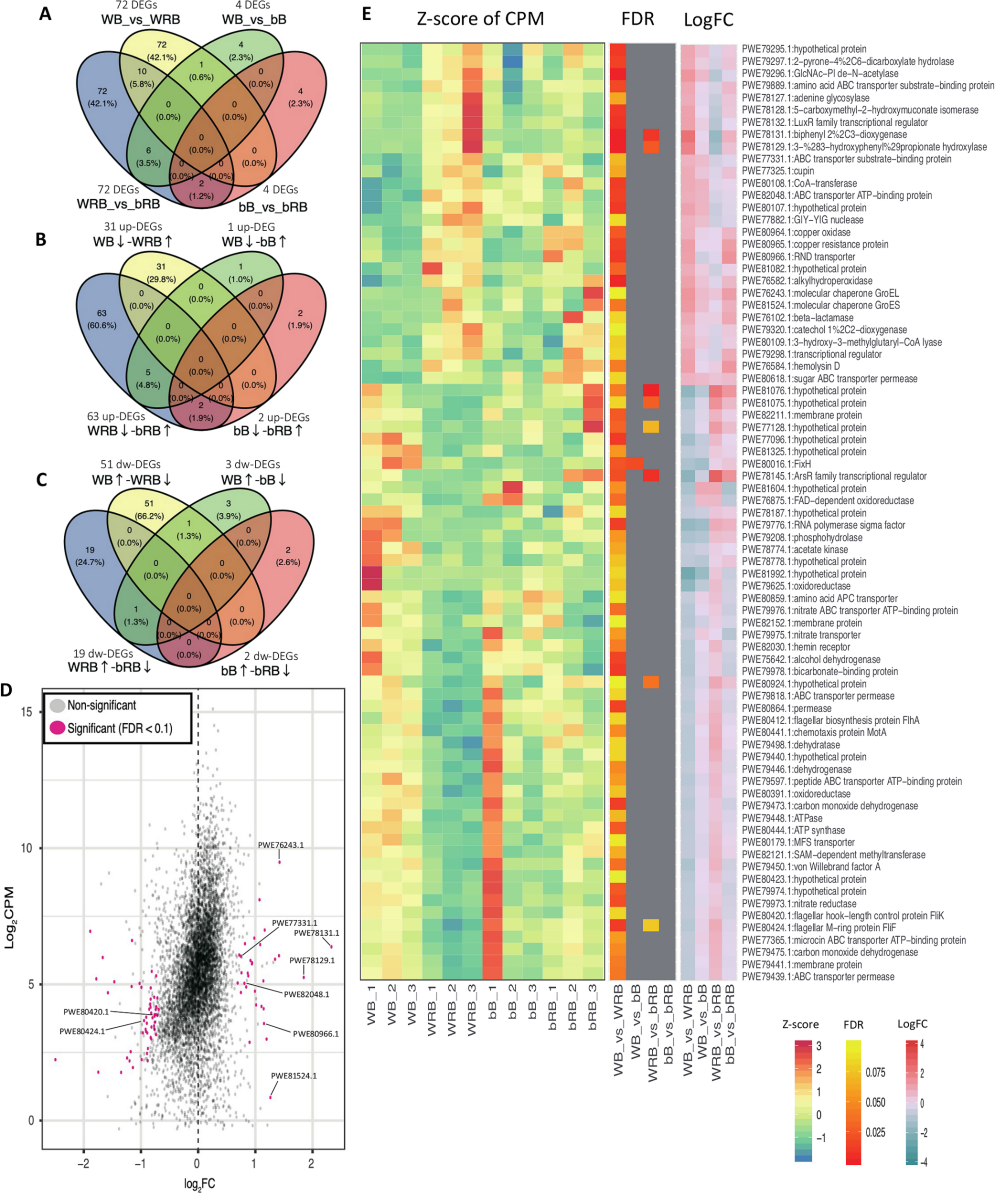
Differentially expressed genes (DEGs) of SUTN9-2 WT versus Δ*bclA* in response to BNM-B (WB and bB) and BNM-B+rice extract (*indica*) (WRB and bRB). Venn diagram of all DEGs of WB_vs_WRB, WB_vs_bB, WRB_vs_bRB, and bB_vs_bRB (A), showing up-regulated genes (B), and down-regulated genes (C). MA plot of up- and down-regulated DEGs of WB_vs_WRB and genes of interest were labeled (D). Expression pattern of DEGs of WB, bB, WRB, and bRB. FDR (≤0.1) and logFC of DEGs of WB_vs_WRB, WB_vs_bB, WRB_vs_bRB, and bB_vs_bRB. The color scale bars are for normalized expression (E).

To analyze the RNAseq transcriptome, total RNA was purified from SUTN9-2 incubated in BNM-B medium in the presence or absence of rice extract. The main reason for performing the transcriptome analysis was to elucidate the mechanisms and factors involved in cell differentiation and nitrogen fixation by endophytic SUTN9-2 in rice plants. The results obtained showed that the expression of a large number of genes was significantly altered (FDR value ≤0.1) in response to rice extract, with the differential expression of 365 genes being significant, representing 63.8% of DEGs (Fig. 3A). The percentage of DEGs was higher (42.1%) in BNM-B with rice extract-treated SUTN9-2 WT cells than in BNM-B-treated cells (Fig. 3A). The effects of Δ*bclA* in rice extract were also demonstrated because 42.1% of genes were differentially regulated in treated WT SUTN9-2 cells in the BNM-B with rice extract group compared to Δ*bclA* in the BNM-B with rice extract group (Fig. 3A). These results revealed the influence of rice extract and the *bclA* gene on the expression of genes in SUTN9-2.

The highest differentially up-regulated genes in SUTN9-2 WT in response to rice extract were biphenyl-2,3-diol 1,2-dioxygenase (PWE78131.1; 2.33 logFC) followed by 3-(3-hydroxyphenyl) propanoate hydroxylase (PWE78129.1; 1.85 logFC) (Fig. 3D and E). Both of these genes belong to the class of oxidoreductase catalytic enzymes involved in the degradation of plant-related compounds and interact with oxygen atoms, which are incorporated into the substrate. The third and fifth highest differentially up-regulated genes were the molecular chaperones sGroEL (PWE76243.1; 1.42 logFC) and GroES (PWE81524.1; 1.34 logFC), which are required for the proper folding of many proteins and the function of the nitrogen fixation regulatory protein NifA. The results from the RNAseq analysis indicated that the differential expression of genes involved in nitrogen fixation and nitrogen metabolism in response to rice extract was not significant (Fig. 3E), whereas qRT-PCR results showed that nitrogen fixation genes (*nifH* and *nifV*) were up-regulated (Fig. 2B) because their expression was detected in rice plants (Piromyou *et al.*, 2017; Greetatorn *et al.*, 2019). However, a key difference between the two experiments needs to be considered. The experimental set-up for the transcriptome analysis was performed on a bigger scale than that for qRT-PCR in order to obtain a sufficiently large number of SUTN9-2 cells in rice extract for RNA purification and the transcriptome analysis. This may have been affected by the different oxygen levels in the two experimental set-ups, which perturbed *nif* gene expression in the transcriptome experiment.

DEGs in response to rice extract were also found to code for a protein involved in the cationic peptide transport system ATP-binding process. These DEGs include genes involved in the ABC transporter ATP-binding protein (PWE82048.1; 0.84 logFC) and ABC transporter substrate-binding protein (PWE77331.1; 7.5 logFC). The gene coding for efflux pumps was also found to be differentially up-regulated in the presence of rice extract (Fig. 3D and E). This gene belongs to the resistance nodulation and cell division family (RND) efflux system (PWE80966.1; 1.15 logFC), which may be involved in bacterial defenses against toxic plant metabolites. These results suggested the presence of cationic antimicrobial peptide (CAMP) in rice plants, which is toxic and affects bacterial cell differentiation. The significantly down-regulated DEGs of SUTN9-2 WT in the response to rice extract were related to flagella (PWE80412.1, PWE80420.1, and PWE80424.1) (Fig. 3E). In contrast, these genes were up-regulated in SUTN9-2 Δ*bclA* (PWE80424.1) in response to rice extract (Fig. 3D), indicating the influence of rice extract and Δ*bclA* on cell motility.

### qRT-PCR validation

**Fig. 4.**
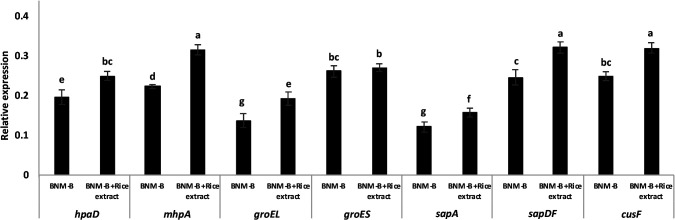
qRT-PCR analysis data for significant DEGs in SUTN9-2 WT between BNM-B alone and BNM-B with rice extract (*indica*). Significance at *P*≤0.05 is indicated by the mean±standard deviation (*n*=3).

qRT-PCR was performed for DEGs, including *hpaD*, *mhpA*, *groEL*, *groES*, *sapDF*, *sapA*, and *cusF* in SUTN9-2 WT that significantly differed between the BNM-B with rice extract (*indica*) group and BNM-B alone group. The results obtained showed that the majority of genes were up-regulated in response to rice extract, which were similar to those from the transcriptome analysis. The expression of *mhpA* appeared to be more strongly up-regulated in response to rice extract (0.09-fold), followed by *sapDF* (0.07-fold), *cusF* (0.07-fold), *groEL* (0.06-fold), *hpaD* (0.05-foid), *sapA* (0.04-fold), and *groES* (0.01-fold), respectively (Fig. 4).

## Discussion

The present results indicate that SUTN9-2 undergoes major changes in cell size and nitrogen fixation efficiency in response to rice extract (Fig. 1). The significant increase observed in the elongated cell size of SUTN9-2 was associated with more efficient nitrogen fixation. Similarly, a TBD process occurs before effective nitrogen fixation is established. These bacteroids are enlarged and polyploid and have lost their capacity to produce progeny (Mergaert *et al.*, 2006; Alunni and Gourion, 2016). The small bacteroid size (1–2.5‍ ‍μm) in alfalfa (*Medicago sativa*) nodules had a low nucleic acid content and did not exhibit acetylene reduction activity (4.9‍ ‍μmol C_2_H_2_ reduced [10^10^]^–1^ bacteroids). The enlarged bacteroid size (5–7‍ ‍μm) had a high nucleic acid content and was very active for acetylene reduction (83.3‍ ‍μmol C_2_H_2_ reduced [10^10^]^–1^ bacteroids) (Paau and Cowles, 1978). The bacteroids of bradyrhizobia in *A. afraspera* and *A. indica* exhibit differentiation along with a high DNA content, such that the mean DNA content of free-growing *Bradyrhizobium* sp. ORS285 was 1 to 2 genome complement (1C to 2C) and that of the bacteroid was 7C to 16C ploidy levels (Czernic *et al.*, 2015; Guefrachi *et al.*, 2015).

**Fig. 5.**
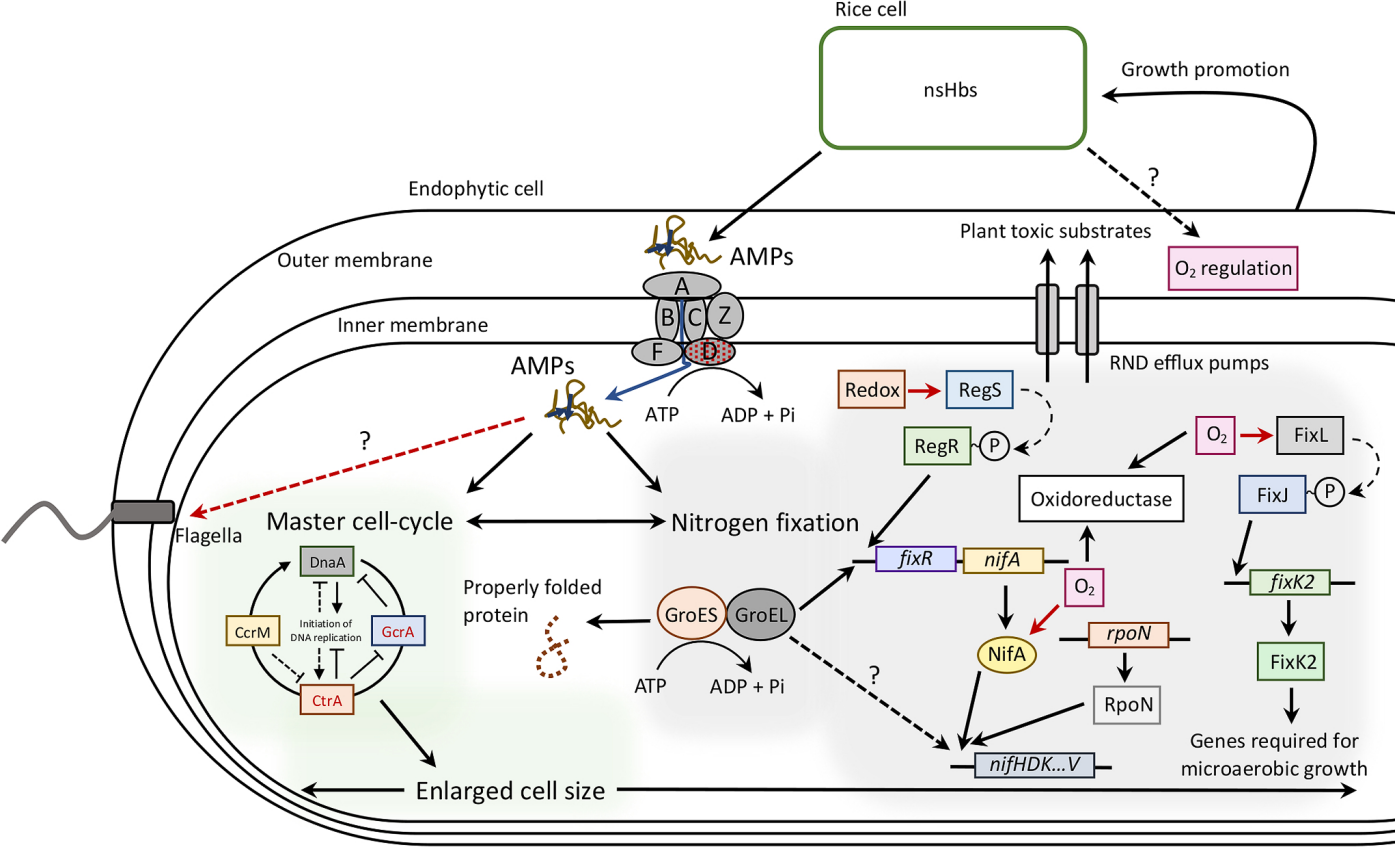
Working model of cell differentiation and nitrogen fixation by SUTN9-2 exposed to rice extract. The currently implied AMPs from rice plants are predictably recognized by BclA ABC transporters belonging to Sap ABC transporter family-like AMP recognition receptors. AMPs are targeted to the cell membrane of bradyrhizobia, localizing the BclA or Sap transporter, promoting the import of AMPs and providing protection against the antimicrobial activity of these peptides. AMPs affect master cell cycle regulators by reducing GcrA activity and the CtrA autoregulation pathway, but promoting DnaA and CcrM for the initiation of DNA replication, resulting in an increase in DNA content and enlarged cell size. A defect in flagellar activity during cell differentiation was observed. This was followed by increased nitrogen fixation activity, which correlated with an enlarged cell size. Excess oxygen perturbs the nitrogen regulatory protein NifA and reduces nitrogen fixation activity. Oxidoreductase may play a role in controlling oxygen to an appropriate level for nitrogenase activity. GroESL co-regulated with the nitrogen regulatory gene *nifA* and *rpoN* RNA polymerase for the formation of a functional nitrogenase. The successful colonization of bradyrhizobia in rice plants is triggered by the RND efflux system and induced rice nsHbs then regulate low oxygen partial pressure and facilitate nitrogen-fixing endophytic cells.

Cell differentiation by *Bradyrhizobium* bacteroids occurred in the interaction with defensin-like antimicrobial peptides (DEFs), which are found in the IRLC and Dalbergioids legume families with the action of BclA transporters (Guefrachi *et al.*, 2015). Based on a phylogenetic tree of SbmA_bacA domain proteins, three genes of *Bradyrhizobium* sp. strain ORS285 were revealed. Two genes (BRAO285v1_250005 and BRAO285v1_950010) are in the *Bradyrhizobium* homologous clade and one (BRAO285v1_1320006) in the BclA clade (Guefrachi *et al.*, 2015). However, only the mutant in gene BRAO285v1_1320006 had abnormal (undifferentiated) bacteroid cells, and exhibited markedly reduced nitrogen fixation activity in the host plants of *Aeschynomene* spp. (Guefrachi *et al.*, 2015). Similarly, SUTN9-2 Δ*bclA* in the present study belonged to the BclA clade. This gene was disrupted according to high homology with the BclA of ORS285 (Fig. S6). Based on the KO (KEGG [Kyoto Encyclopedia of Genes and Genomes] Orthology) annotation in the reconstruction pathway, the ABC transporter involving the CAMP resistance of SUTN9-2 Δ*bclA* was perturbed by the deletion of the ABC transporter ATP-binding domain (*bclA*; PWE81210.1) belonging to SapDF (Lopez-Solanilla *et al.*, 1998). However, the transcriptomic analysis showed that the ABC transporter substrate-binding protein consisting of SapA (PWE77331.1) and ABC transporter ATP-binding protein consisting of SapDF (PWE82048.1) were significantly and differentially up-regulated in response to rice extract (Fig. 3D and E, Table S2). A phylogenetic analysis based on the sequences of SbmA_BacA domain proteins showed that SapA (PWE77331.1) and SapDF (PWE82048.1) identified in SUTN9-2 were distant from the BclA and *Bradyrhizobium* homologous clades identified in *Bradyrhizobium* spp. However, both of these genes showed 97% similarity to these clades (Fig. S6) (Guefrachi *et al.*, 2015). These results indicate that SapA (PWE77331.1) and SapDF (PWE82048.1) play an important role together with SUTN9-2 Δ*bclA* (PWE81210.1; data not shown) for cell differentiation, nitrogen fixation in symbiosis, and responses to rice extract because the cell differentiation of SUTN9-2 Δ*bclA* was still maintained in both systems of bacteroids in legume plants (data not shown) and bacteria in response to rice extract (Fig. S2 and S4). However, decreases were also observed in the elongated cell sizes and nitrogen fixation activities of Δ*bclA* and Δ*nifV*. These results indicated the effects of *bclA* and *nifV* genes on the cell size and nitrogen fixation activity of SUTN9-2 cells in response to rice extract. The *sap* mutant pathogenic bacterium *Erwinia chrysanthemi* was sensitive to killing by antimicrobial peptides (AMPs) (wheat α-thionin and snaking-1) from potato tubers (Lopez-Solanilla *et al.*, 1998). The Sap transporter consists of SapABCDFZ, which shares homology to the ‘ATP-binding cassette’ (ABC) family of transporters that show diverse substrate binding and uptake (Hiles *et al.*, 1987; Abouhamad *et al.*, 1991; Parra-Lopez *et al.*, 1993). SapA is predicted to function as a periplasmic solute-binding protein; SapB and SapC as inner membrane permease proteins; SapD and SapF as ATPase subunits; whereas the function of SapZ remains unknown (Mason *et al.*, 2006). Moreover, previous studies identified several genes encoding cysteine-rich peptides (CRPs), also suggesting several uncharacterized AMPs in rice plants (Silverstein *et al.*, 2007), and genes encoding DEFs were also detected in rice plants (Tantong *et al.*, 2016; Li *et al.*, 2017). These results implied the production of DEFs in rice plants interacting with the Sap transporter of SUTN9-2, which affected enlarged cell sizes and high DNA contents in SUTN9-2 in response to rice extract (Fig. 5), similar to the cell differentiation and polyploidy of bacteroids in legume plants affected by NCR peptides.

Moreover, the gene coding for RND transporter efflux pumps (PWE80966.1) was differentially up-regulated in SUTN9-2 WT exposed to rice extract (Fig. 3D and E). Efflux pumps are transport proteins involved in the extrusion of toxic substrates into the external environment (Coutinho *et al.*, 2015). The RND efflux system has been recognized to play an important role in the successful colonization of the apple tree by the phytopathogen *Erwinia amylovora* (Burse *et al.*, 2004). RND efflux systems are strongly up-regulated in *B. kururiensis* M130 in the presence of rice extract (Coutinho *et al.*, 2015). This type of efflux system may be important in bacterial survival defenses against toxic-plant metabolites, including DEFs (Fig. 5).

Flagellar biosynthesis is mostly down-regulated in symbiotic bacteroid cells. The transcriptome analysis of *Mesorhizobium loti* revealed that genes for flagellar formation were strongly repressed under the symbiotic condition because rhizobia under this condition do not need to be motile (Uchiumi *et al.*, 2004; Tatsukami *et al.*, 2013). In addition, the relative expression level of the SUTN9-2 flagella biosynthetic protein (*fliP*) in response to rice root exudate was lower than that without rice root exudate. The expression level of *fliP* also slightly decreased with an increase in plant age (Piromyou *et al.*, 2015b). Similarly, the differentially down-regulated expressed genes of SUTN9-2 WT were related to flagellar biosynthesis (PWE80412.1, PWE80420.1, and PWE80424.1) in response to rice extract. In contrast, these genes were up-regulated in Δ*bclA* (PWE80424.1) in response to rice extract (Fig. 3D). This up-regulation has also been observed in transcriptome experiments with *B. kururiensis* in response to rice extract and allows bacteria to escape host defense responses (Coutinho *et al.*, 2015). Therefore, the down-regulation of flagellar biosynthesis appears to be supporting the cell differentiation of SUTN9-2 in response to rice extract, similar to bacteroids in the symbiotic condition. In contrast, Δ*bclA* lacking some parts of the transporter against plant AMPs up-regulate genes for flagellar biosynthesis, thereby allowing bacteria to move faster in the plant environment in order to escape host defense responses (Fig. 5).

Rice extract (*O. sativa* L. ssp. *indica* cv. Pathum Thani 1) affected cell elongation and nitrogen fixation activity in SUTN9-2 more strongly than rice extract (*O. sativa* L. *japonica* cv. Nipponbare) in all treated SUTN9-2 WT, Δ*bclA*, and Δ*nifV* (Fig. 1D, E, F, and S2). Elongated cell size was smaller in Δ*nifV* than in WT and Δ*bclA* SUTN9-2, and also in Δ*bclA* than in WT SUTN9-2 (Fig. 1D, E, and S2). When SUTN9-2 WT was inoculated into the Thai rice cultivar Pathum Thani 1 (*indica*) and Japanese rice cultivar Nipponbare (*japonica*), the number of SUTN9-2 WT cells at 30 dpi was higher in Thai rice root tissues (*indica*) than in Japanese rice (*japonica*), with 10^3^ and 10^1^ CFU g^–1^ root fresh weight, respectively (Piromyou *et al.*, 2015b). The population density of SUTN 9-2 was larger in Thai rice (*indica*) than in Japanese rice (*japonica*). In addition, Thai bradyrhizobial strain SUTN9-2 was suggested to promote the total dry weight of rice (*indica*) more effectively than Japanese bradyrhizobial strains (Piromyou *et al.*, 2015a). On the other hand, rice (*indica*) responded positively only to putative Thai rice endophytic bradyrhizobia, while this phenomenon was not observed in Japanese rice (*japonica*) (Piromyou *et al.*, 2015a, 2015b). In addition, the type III secretion system (T3SS) of SUTN9-2 is involved in bradyrhizobial infections in rice plants. The density of the SUTN9-2 T3SS mutant in Thai rice (*indica)* was significantly lower than that of SUTN9-2 WT, and this property was not detected in Japanese rice (*japonica*), indicating SUTN9-2 had the ability to overcome native host rice defense responses through the function of T3SS and also that rice developed a system to protect itself from non-native soil bacteria (Piromyou *et al.*, 2015b). These results imply that the rice cultivar and bacterial strain are important factors that control the compatibility of the rice-bacterium relationship, which may contribute to bradyrhizobia-host evolution (Piromyou *et al.*, 2015b). These results support the existence of a preferable host for SUTN9-2 in rice species, which may also contribute to greater increases in the cell size enlargement and nitrogen fixation activity of SUTN9-2 in response to rice extract from *indica* than to that from *japonica*.

The attenuated pattern of *ctrA* expression in NCR-treated cells during the cell cycle may be caused by reduced GcrA activity or by defects in the CtrA autoregulation pathway. DnaA appears to be necessary for repeated initiation rounds of DNA replication during endoreduplication *in vivo* (Collier, 2012). CcrM DNA methyltransferase is present and active for a short time and essential for methylation at the start of DNA replication (Collier, 2012). In the present study, the results obtained suggested that AMPs in rice extract affect master cell-cycle regulators by suppressing the expression of *ctrA* and promoting *dnaA* and *ccrM* expression, resulting in an increase in cell size and DNA content (Fig. 2A, 5, and S3). In addition, the *nifV* gene may affect cell size enlargement following exposure to rice extract. The expression of genes involved in the nitrogen fixation activity (*nifH* and *nifV*) of SUTN9-2 has been detected in the endophytic relationship with rice plants (Piromyou *et al.*, 2017; Greetatorn *et al.*, 2019). Furthermore, the expression of *nifH*, *nifV*, and *bclA* in WT and Δ*bclA* SUTN9-2 increased in response to rice extract. In contrast, the expression of these genes in SUTN9-2 Δ*nifV* decreased in response to rice extract (Fig. 2B). These results suggest similarities in the expression model between these genes and the genes involved in the master cell cycle following exposure to rice extract. Furthermore, the effects of the interaction between the rice extract and BclA transporter on cell differentiation and nitrogen fixation were implied based on the expression of cell cycle and *nif* genes in response to rice extract (Fig. 5). Non-symbiotic hemoglobins (nsHbs) have been detected in several monocot plants. Rice (*O. sativa*) contains five copies of the *nsHb* gene, namely, *hb1*-*hb5* (Lira-Ruan *et al.*, 2002). *hb1*, *hb2*, and *hb5* are expressed in rice embryonic organs and vegetative organs, and their appear to function as oxygen carriers or in some aspects of oxygen metabolism (Garrocho-Villegas *et al.*, 2008; Lira-Ruan *et al.*, 2011). Hormone and stress response promoters exist upstream of the rice *hb5* gene, which was transcribed in rice organs. The amino acid sequence and protein model structure of Hb5 differ from those of rice Hbs 1 to 4 (Garrocho-Villegas *et al.*, 2008), suggesting different expression levels between *hb1* and *hb5* (Fig. 2C). However, they are present at very low levels inside host rice cells. In addition, the physiological functions of rice nsHbs are not involved in oxygen transport, but more closely resemble known oxygen sensors (Goodman and Hargrove, 2001). This finding revealed that nsHbs in rice plants may function as a regulator to maintain low oxygen partial pressure for nitrogenase activity to facilitate nitrogen-fixing endophytic cells (Fig. 5).

The RNAseq experiment provided a global view of the gene expression profile in response to rice extract. RNAseq results indicated that SUTN9-2 endophytic cells were affected by rice extract and the Δ*bclA* mutation, which is consistent with the results for significant DEGs (Fig. 3A). The expression of genes involved in the cell cycle and nitrogen fixation was up-regulated when SUTN9-2 was exposed to rice extract. These results are in contrast to those obtained from the transcriptome analysis of SUTN9-2 in the presence of rice extract. These genes did not show significant DEGs in the response to rice extract. Bacteria capable of utilizing biphenyl and phenylpropanoid compounds as carbon and energy sources are widely distributed in natural environments, and may originate from the putrefaction of proteins in soil or as breakdown products of several constituents of plants, such as lignin, various oils, and resins (Ferrandez *et al.*, 1997; Díaz *et al.*, 1998; Wesche *et al.*, 2005). The highest up-regulated DEGs involved in biphenyl-2,3-diol 1,2-dioxygenase (PWE78131.1) and 3-(3-hydroxyphenyl) propanoate hydroxylase (PWE78129.1) were observed. These genes belong to the class of oxidoreductases acting on donors with oxygen and the incorporation of atoms of oxygen into the substrate (Díaz *et al.*, 1998; Wesche *et al.*, 2005), which may have a role in controlling oxygen to an appropriate level for nitrogenase activity (Fig. 3D and E). This result suggests excess oxygen in the experimental set-up for the transcriptome analysis (50-mL tube), which affects nitrogenase activity. A larger scale experiment (50-‍mL tube) was performed to obtain a large amount of SUTN9-2 cells in rice extract for the transcriptome analysis, more than the experimental set-up for qRT-PCR (10-‍mL tube). The nitrogenase enzyme complex is highly sensitive to molecular oxygen, which irreversibly inactivates the enzyme. The inhibition of *nif* gene expression by molecular oxygen at the nitrogen regulatory protein NifA post-transcriptional stage was detected in *B. japonicum* (Fischer and Hennecke, 1987; Kullik *et al.*, 1989). Therefore, the suppression of nitrogen fixation genes observed in the transcriptome analysis may be due to the effects of excess oxygen on nitrogenase sensitivity (Fig. 5).

The molecular chaperones GroEL (PWE76243.1) and GroES (PWE81524.1) were identified as significant differentially up-regulated genes in response to rice extract (Fig. 3D and E). GroESL chaperonins are required for the formation of a functional nitrogenase in *B. japonicum*, which is co-regulated together with the symbiotic nitrogen regulatory gene *nifA* and transcribed by σ^54^ RNA polymerase (Fischer *et al.*, 1993, 1999). However, the requirement of chaperonins for nitrogen fixation does not occur at the level of RegSR-NifA-σ^54^- or FixLJ-FixK-dependent gene regulation (Fischer *et al.*, 1999). This finding indicated that the nitrogen fixation of SUTN9-2 in response to rice extract may be affected by GroESL chaperonins with or without Nif-dependent gene regulation. Collectively, these studies imply that the nitrogen fixation of SUTN9-2 was induced in response to rice extract because the differentially up-regulated genes of GroESL chaperonins were detected (Fig. 5).

Based on the results and transcriptomic findings reported herein, a proposed model for the cell differentiation and nitrogen fixation activity of SUTN9-2 in response to rice extract was shown (Fig. 5). Rice plants are predicted to produce AMPs that are recognized during interactions by AMP recognition receptors (Sap ABC transporter family), thereby promoting the import of AMPs and protecting SUTN9-2 cells against the antimicrobial activity of these peptides. Following the recognition and transduction of these AMPs, several DEGs are induced in SUTN9-2. These AMPs predictably modulate master cell cycle regulators, thereby causing cell differentiation. These interactions induced several processes, including oxidoreductase, GroESL chaperonin, the RND efflux system, and flagellar biosynthesis, which may promote cell size enlargement, nitrogen fixation, and, ultimately, rice growth (Fig. 5). These results imply similarities in the mechanisms and factors involved in cell differentiation and nitrogen fixation between endophytic cells in rice plants and symbiotic cells in legume plants, which are based on similar mechanisms from both the bacterial side (BclA-like transporters) and plant side (AMPs). It is important to understand the mechanisms underlying the regulation of the factors, molecules, and signals of the plant as well as the bacterial cells involved in inducing cell differentiation and nitrogen fixation in endophytic cells required for *in-planta* survival and plant growth promotion. The specialized legume plant genes involved in symbiotic interactions may have arisen from a pre-existing non-symbiotic plant gene, such as rice plants, suggesting convergent coevolution in these distant plant species.

## Citation

Greetatorn, T., Hashimoto, S., Maeda, T., Fukudome, M., Piromyou, P., Teamtisong, K., et al. (2020) Mechanisms of Rice Endophytic Bradyrhizobial Cell Differentiation and Its Role in Nitrogen Fixation. *Microbes Environ ***35**: ME20049.

https://doi.org/10.1264/jsme2.ME20049

## Supplementary Material

Supplementary Material
