# Supplementary material for: Mechanisms of Rice Endophytic Bradyrhizobial Cell Differentiation and Its Role in Nitrogen Fixation
Source: Microbes Environ. 2020 Jul 29;35(3):ME20049. doi: 10.1264/jsme2.ME20049 (PMC7511792; doi:10.1264/jsme2.ME20049)
Supplement: Supplementary file 1 — Supplementary Material [file 35_20049_s1.pdf]

Table S1. Primers used in this study.

Target name by type	Primer name	Gene description	Primer sequence (5'→3')	Description of design and reference
House keeping				
dnaK	dnaK-9-2/858F	70 kDa chaperone protein	tcacgacttcctggaccttc	Designed from *dnaK* of SUTN9-2 (LAXE00000000)
	dnaK-9-2/1077R		atcaacctgcccttcatcac	
EF-1α	EF-1α-Os03g08020F	Elongation factor 1α	gtcattggccacgtcgactc	Caldana et al. (2007)
	EF-1α-Os03g08020R		tgttcatctcagcggcttcc	
Nitrogen fixation				
nifH	nifH-9-2/489F	Dinitrogenase reductase	taacatctccaagggcatcc	Designed from *nifH* of SUTN9-2 (LAXE00000000)
	nifH-9-2/758R		ccgccattattgtgaacctt	
nifV	nifV-9-2/850F	Homocitrate synthesis	cgtgcgattcctatgaacaa	Designed from *nifV* of SUTN9-2 (LAXE00000000)
	nifV-9-2/1107R		gatggcatatttgcggactt	
ABC trasporter				
bclA	bclA-9-2/349F	BclA-ABC transporter ATP-binding domain	cactatcggatgcagctcaa	Designed from *bclA* of SUTN9-2 (LAXE00000000)
	bclA-9-2/639R		gagattgacgagaggcgaac	
Master cell-cycle				
gcrA	gcrA-9-2/106F	Cell cycle regulator	gccgcagaagaagaaatcc	Designed from *gcrA* of SUTN9-2 (LAXE00000000)
	gcrA-9-2/3087R		agcatatgatgcgggtgac	
ctrA	ctrA-9-2/372F	Cell cycle transcriptional regulator	acggcttggtgagataatcg	Designed from *ctrA* of SUTN9-2 (LAXE00000000)
	ctrA-9-2/607R		gggctacgaaatcctggaag	
dnaA	dnaA-9-2/709F	Chromosomal replication initiator	gagatcgtcgatgacaagca	Designed from *dnaA* of SUTN9-2 (LAXE00000000)
	dnaA-9-2/923R		tcaacccgctctacatccat	
ccrM	ccrM-9-2/329F	DNA methyltransferase	gccagttcatcgaaaagctc	Designed from *ccrM* of SUTN9-2 (LAXE00000000)
	ccrM-9-2/557R		agacggtgcggaattaacag	
Hemoglobin				
hb1	hb1-indica/341F	Hemoglobin1	catgtccgtcttcgtcatgg	Designed from *hb1* of *O. sativa* L. ssp. *indica* (U76029)
	hb1-indica/500R		ttgagggtggtgtctctcac	
hb5	hb5-indica/456F	Hemoglobin5	gtcttcgtcatggcaagcaa	Designed from *hb5* of *O. sativa* L. ssp. *indica* (EF061459)
	hb5-indica/594R		ctccctcaccttcacgtcac	
qRT-PCR validation				
hpaD	hpaD-9-2/15F	Biphenyl 2,3-dioxygenase	gctcggctatatcggaatca	Designed from *hpaD* of SUTN9-2 (LAXE00000000)
	hpaD-9-2/233R		cctgccagcttatccagttc	
mhpA	mhpA-9-2/688F	3-3-hydroxyphenyl propionate hydroxylase	ccgaatggaacgtgtagacc	Designed from *mhpA* of SUTN9-2 (LAXE00000000)
	mhpA-9-2/906R		acgacctcggtgattacagc	
groEL	groEL-9-2/536F	Molecular chaperone GroEL	tcgtagtccgaggtggtttc	Designed from *groEL* of SUTN9-2 (LAXE00000000)
	groEL-9-2/750R		gaggatatcgcgatccttacc	

Table S1. (Continued).

Target name by type	Primer name	Gene description	Primer sequence (5'→3')	Description of design and reference
groES	groES-9-2/37F	Molecular chaperone GroES	ccgagaagatcgctttcctt	Designed from *groES* of SUTN9-2 (LAXE00000000)
	groES-9-2/241R		gcatcatcattcccgacac	
sapA	sapA-9-2/10F	ABC transporter substrate-binding protein	cgttctgcgacatcgaacta	Designed from *sapA* of SUTN9-2 (LAXE00000000)
	sapA-9-2/241R		cgtcgaaaaccgaaaaatgt	
sapDF	sapDF-9-2/299F	ABC transporter ATP-binding protein	ctcgtccatcagcaggatct	Designed from *sapDF* of SUTN9-2 (LAXE00000000)
	sapDF-9-2/501R		tggagcagaacatcggctat	
cusF	cusF-9-2/92F	RND family efflux transporter	gcgaggtcaagaagatcgac	Designed from *cusF* of SUTN9-2 (LAXE00000000)
	cusF-9-2/296R		gccttctgcatcttggtcac	
bclA mutant construction				
bclA upstream region	up_EcoRI_for	Upstream region of BclA-ABC transporter ATP-binding domain	agcgggaattcgctcaatctgtccgacgatgcg	Designed from *bclA* of SUTN9-2 (LAXE00000000)
	up_ *BamH*I_rev		cctcagcgcgcggatccgatgttgttcacagagctgg	
bclA downstraem region	dw_ *BamH*I_for	Downstream region of BclA-ABC transporter ATP-binding domain	tgtgaacaacatcggatccgcgcgctgaggcgag cgctgg	Designed from *bclA* of SUTN9-2 (LAXE00000000)
	dw_ *Xba*I_rev		cggcggtctagacatgagcgattacg tcttccg	

Table S2. Differentially upregulated gene expression of SUTN9-2 WT in response to rice plant extract by transcriptome analysis.

Protein_ID	Predicted function	logFC	logCPM	*P* value	FDR
PWE78131.1	biphenyl 2, 3-dioxygenase	2.33	6.38	1.24E-12	8.23E-09
PWE78129.1	3-3-hydroxyphenyl propionate hydroxylase	1.85	5.26	3.45E-09	1.07E-05
PWE76243.1	molecular chaperone GroEL	1.42	9.49	0.00	0.09
PWE79296.1	GlcNAc-PI de-N-acetylase	1.41	6.05	4.85E-09	1.07E-05
PWE81524.1	molecular chaperone GroES	1.34	5.92	2.27E-04	0.04
PWE80965.1	copper resistance protein	1.20	2.99	4.14E-05	0.01
PWE76584.1	hemolysin D	1.16	7.00	2.53E-04	0.04
PWE80966.1	RND transporter	1.15	3.56	1.93E-05	0.01
PWE78132.1	LuxR family transcriptional regulator	1.14	4.11	6.92E-06	0.01
PWE76582.1	alkylhydroperoxidase	1.14	5.13	7.73E-07	0.00
PWE78128.1	5-carboxymethyl-2-hydroxymuconate isomerase	1.11	4.19	1.95E-05	0.01
PWE80107.1	hypothetical protein	1.09	6.46	1.14E-05	0.01
PWE79298.1	transcriptional regulator	1.07	8.11	9.09E-04	0.08
PWE79295.1	hypothetical protein	0.99	4.75	1.48E-05	0.01
PWE79889.1	amino acid ABC transporter substrate-binding protein	0.98	6.70	6.40E-05	0.02
PWE79297.1	2-pyrone-4%2C6-dicarboxylate hydrolase	0.94	5.80	4.62E-05	0.01
PWE80108.1	CoA-transferase	0.92	5.88	2.73E-05	0.01
PWE77325.1	cupin	0.90	2.87	0.00	0.06
PWE81082.1	hypothetical protein	0.88	5.31	1.42E-04	0.03
PWE78127.1	adenine glycosylase	0.87	5.41	5.31E-04	0.06
PWE82048.1	ABC transporter ATP-binding protein	0.84	4.91	3.80E-05	0.01
PWE80109.1	3-hydroxy-3-methylglutaryl-CoA lyase	0.82	6.49	7.62E-04	0.08
PWE80964.1	copper oxidase	0.81	5.04	1.01E-04	0.02
PWE77882.1	GIY-YIG nuclease	0.76	5.43	9.81E-04	0.09
PWE77331.1	ABC transporter substrate-binding protein	0.75	6.04	6.17E-04	0.07
PWE80618.1	sugar ABC transporter permease	0.72	6.08	8.02E-04	0.08
PWE79320.1	catechol 1, 2-dioxygenase	0.69	5.05	0.00	0.10

Log_2 fold change (logFC) represent upregulation in the presence of rice extract

Log count per million (logCPM) represent differentially expression degree

Probability value (*P* value)

False discovery rate (FDR) value for RNAseq

Table S3. Total of 865M reads (R1+R2) from the 12 libraries.

Sample	Raw data		
	Fragment #	Read length	Total bases
WB_1	39,626,861	150 x 2	11,888,058,300
WB_2	37,169,231	150 x 2	11,150,769,300
WB_3	31,573,758	150 x 2	9,472,127,400
WRB_1	33,521,269	150 x 2	10,056,380,700
WRB_2	39,326,307	150 x 2	11,797,892,100
WRB_3	32,584,299	150 x 2	9,775,289,700
bB_1	41,851,749	150 x 2	12,555,524,700
bB_2	49,348,149	150 x 2	14,804,444,700
bB_3	38,768,526	150 x 2	11,630,557,800
bRB_1	32,096,739	150 x 2	9,629,021,700
bRB_2	27,820,858	150 x 2	8,346,257,400
bRB_3	29,158,174	150 x 2	8,747,452,200
Total	432,845,920		129,853,776,000

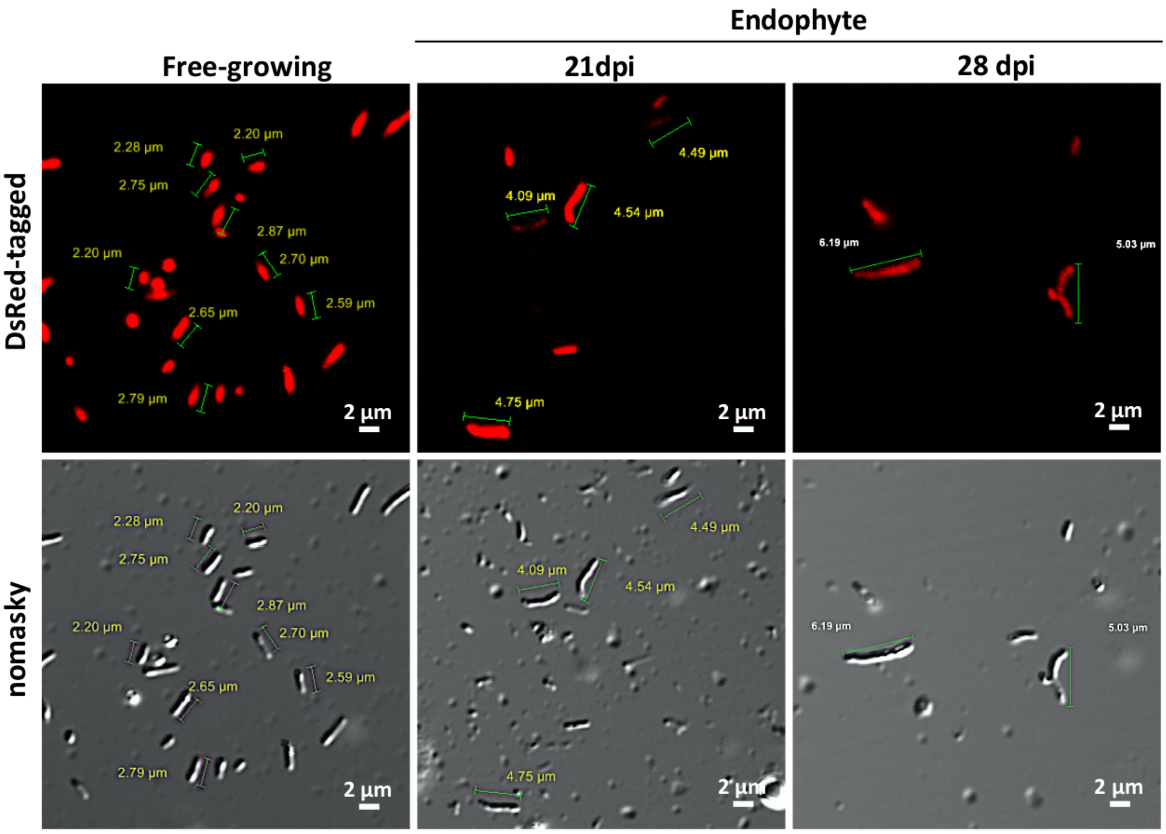

Fig. S1. Cell enlarged size of extracted SUTN9-2 DsRed-tagged at 21 and 28 dpi from rice plant tissues (*indica*) by confocal laser scanning microscope.

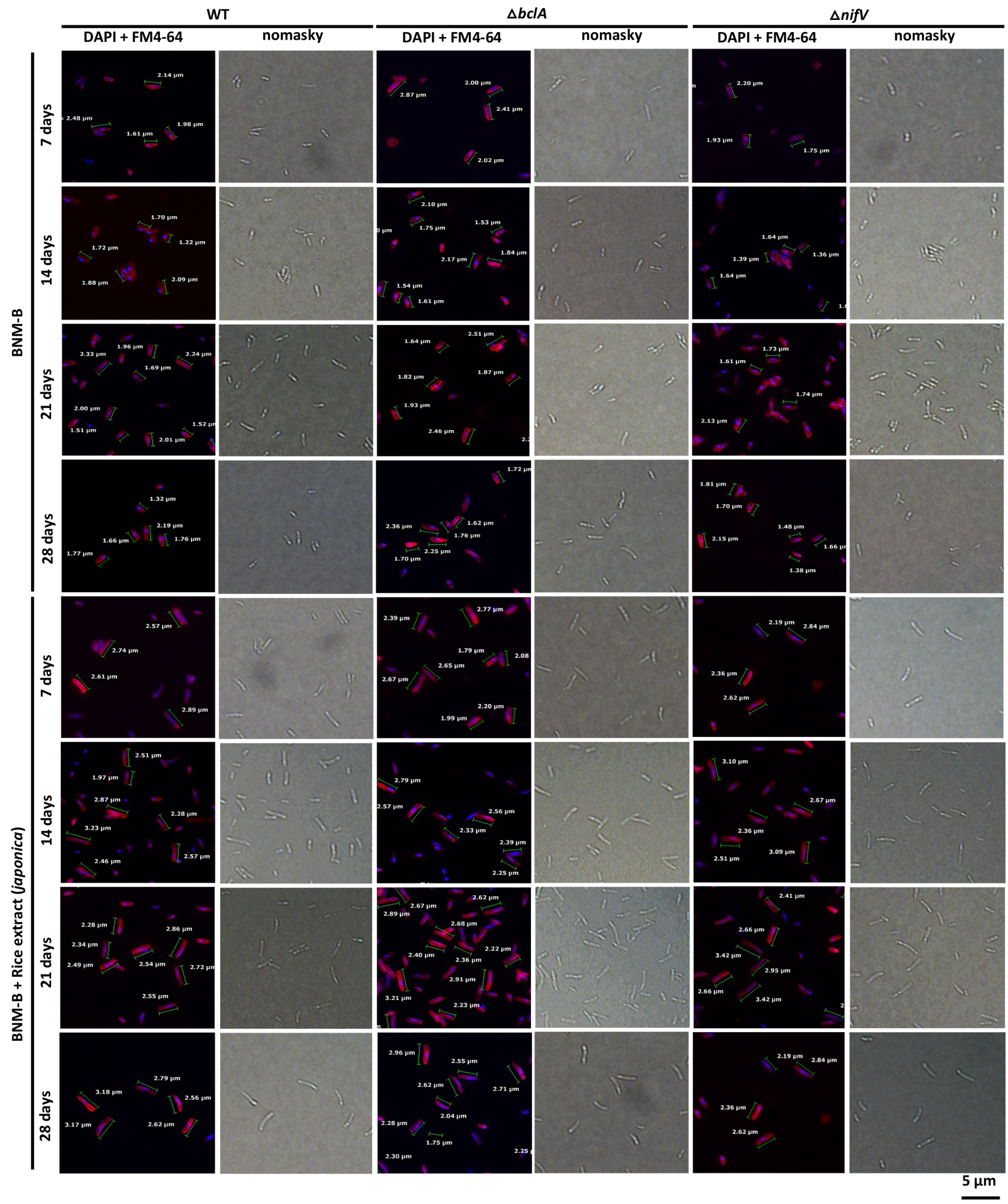

Fig. S2. Cell size and DNA content of SUTN9-2 WT, Δ*bclA*, and Δ*nifV* with FM4-64-stained membrane and DAPI-stained nucleoid in response to BNM-B, BNM-B+rice extract (*indica*), and BNM-B+rice extract (*japonica*) at 7, 14, 21, and 28 days.

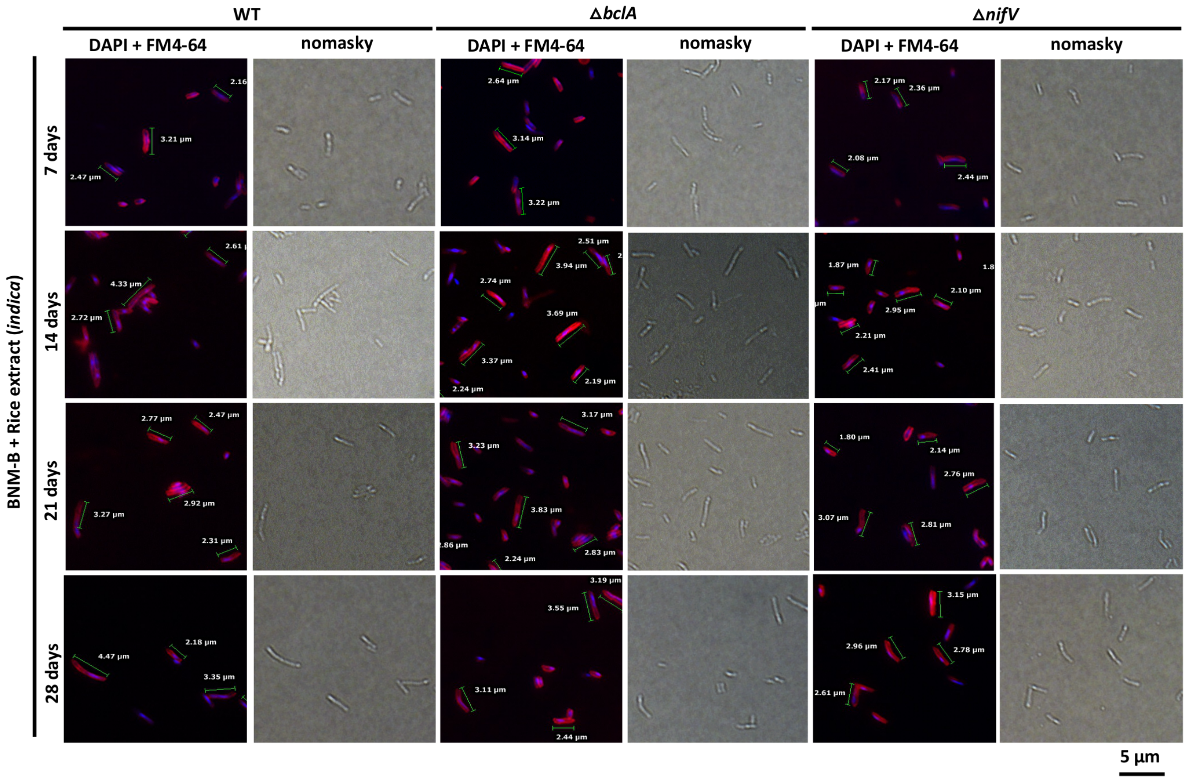

Fig. S2. (Continued)

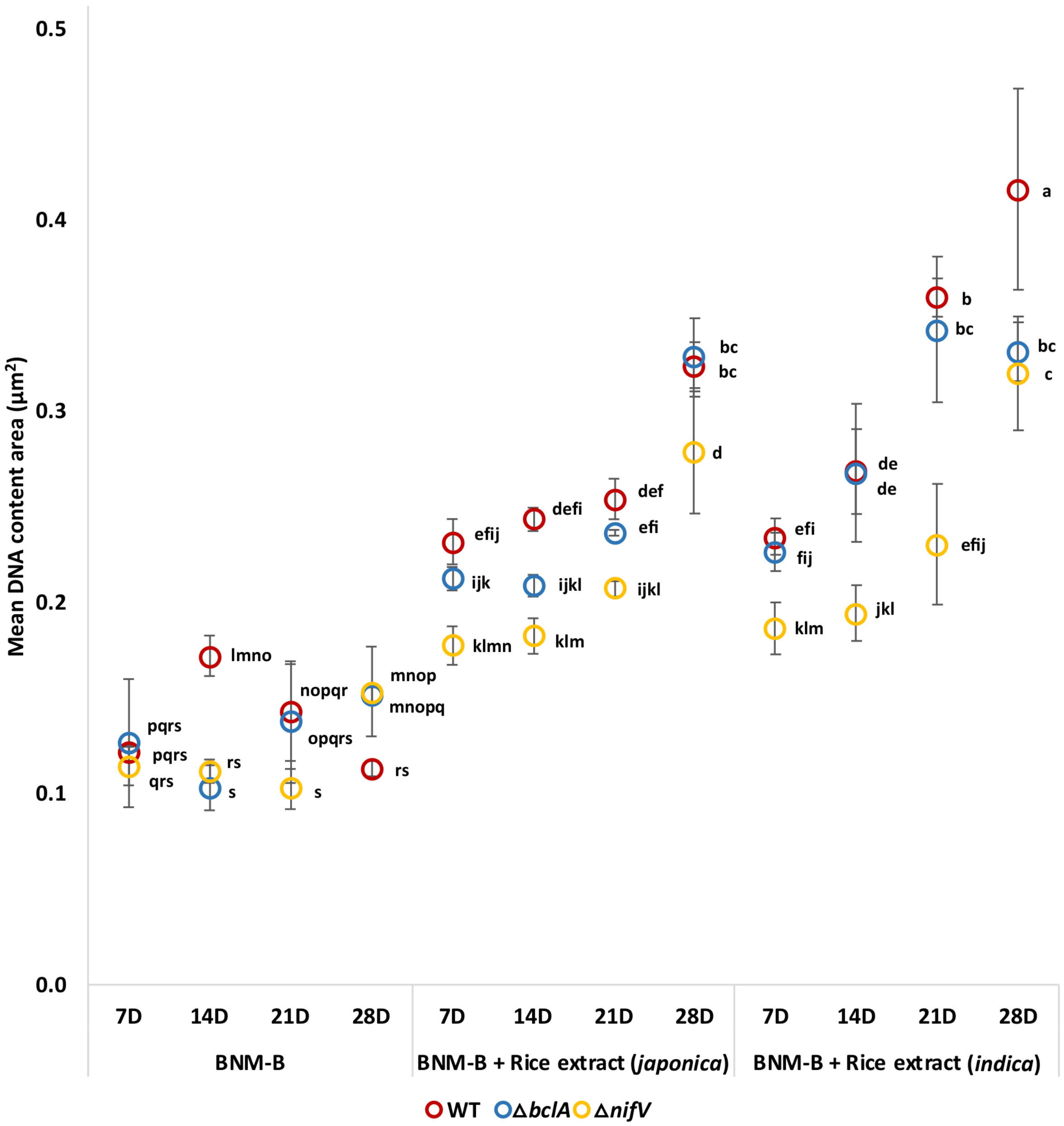

Fig. S3. Mean DNA content areas (µm^2) from DAPI-stained nucleoid of SUTN9-2 WT, Δ*bclA*, and Δ*nifV* in response to BNM-B, BNM-B+rice extract (*indica*), and BNM-B+rice extract (*japonica*) at 7, 14, 21, and 28 days (D), means labeled with different letters are statistically different at a *P* value of ≤0.05 (n=3).

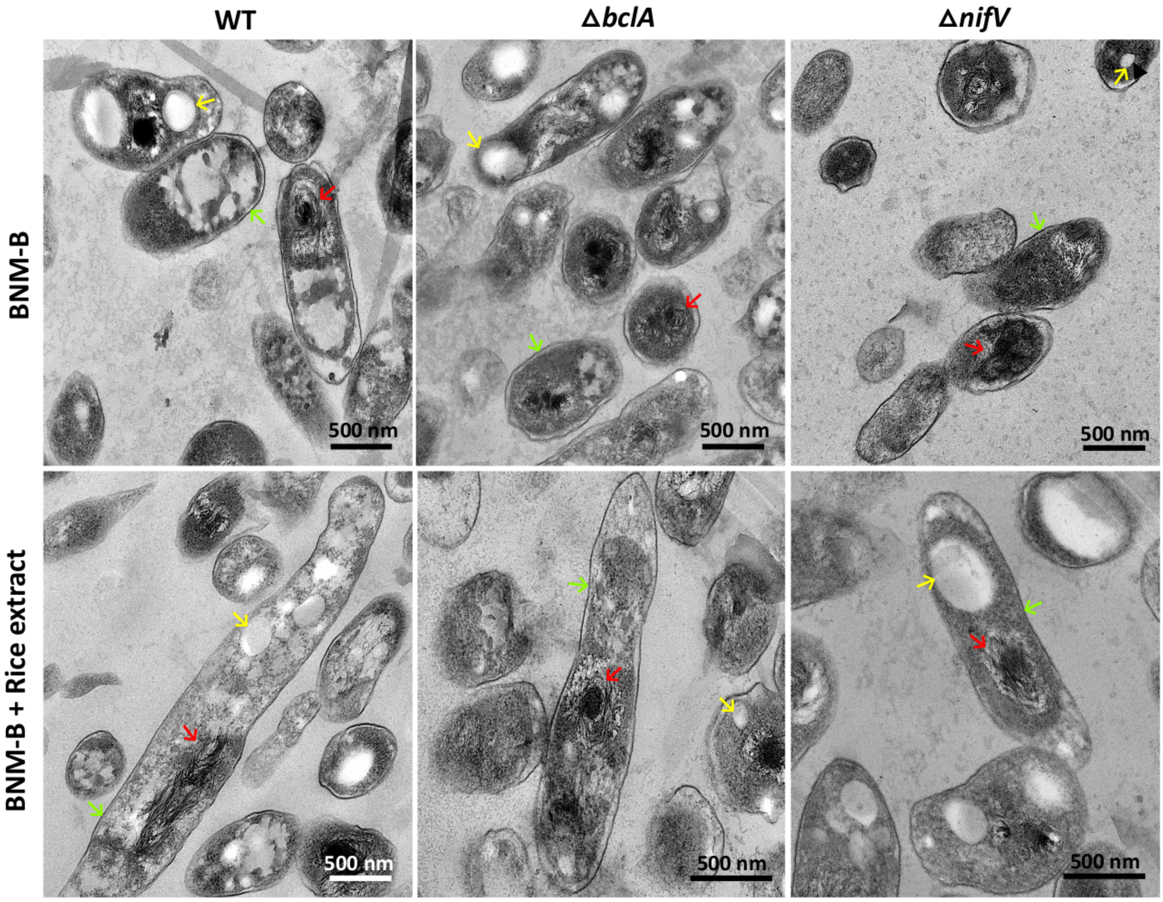

Fig. S4. Cross-section of SUTN9-2 WT, Δ*bclA*, and Δ*nifV* by transmission electron microscope (TEM) in BNM-B and BNM-B+rice extract (*indica*) at 21 days. The enlarged cell size of WT, Δ*bclA*, and Δ*nifV* was observed in response to rice extract. Red arrows mark bradyrhizibial nucleoid, yellow arrows mark bradyrhizibial polyhydroxybutyrate (PHB), and green arrows mask cell membrane.

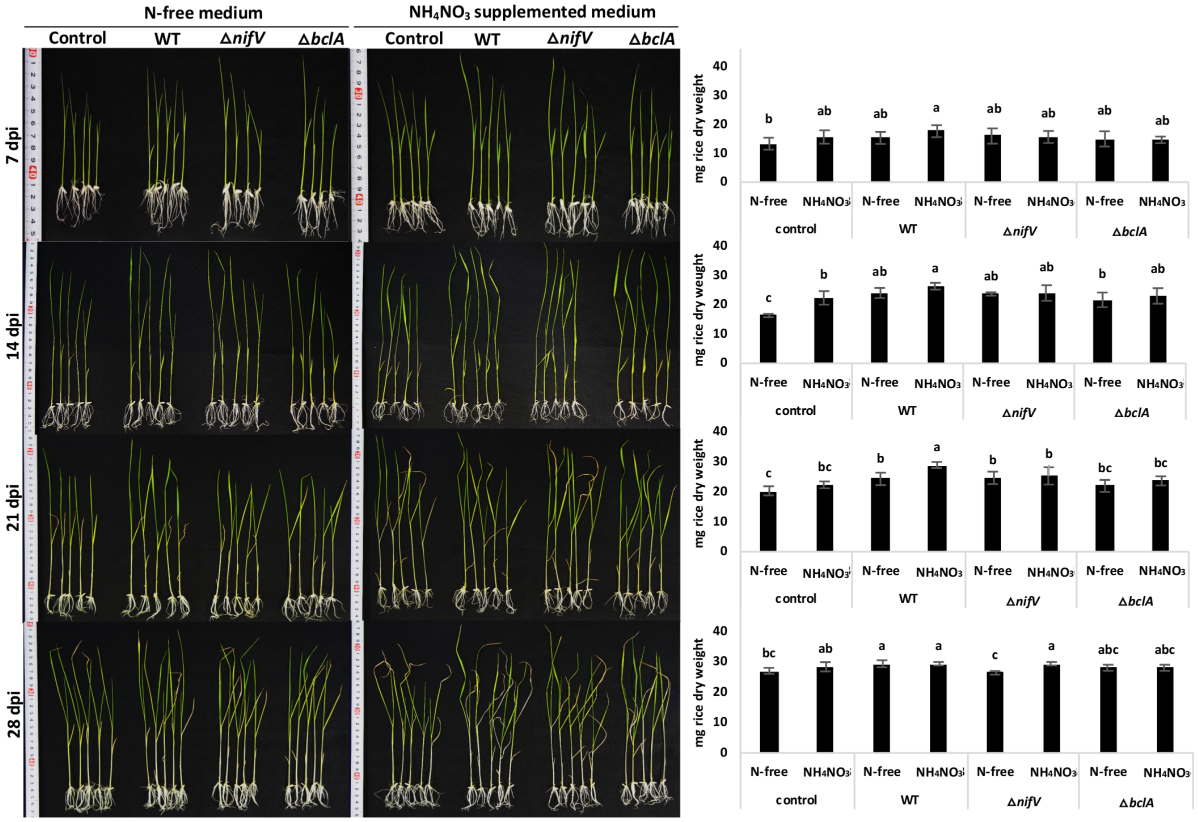

Fig. S5. Effect of SUTN9-2 WT, Δ*bclA*, and Δ*nifV* on rice growth (*indica*) with non-inoculation control in N-free rice medium and N-free supplemented with 1mM ammonium nitrate at 7, 14, 21, and 28 dpi, means labeled with different letters are statistically different at a P value of ≤ 0.05 (n=3).

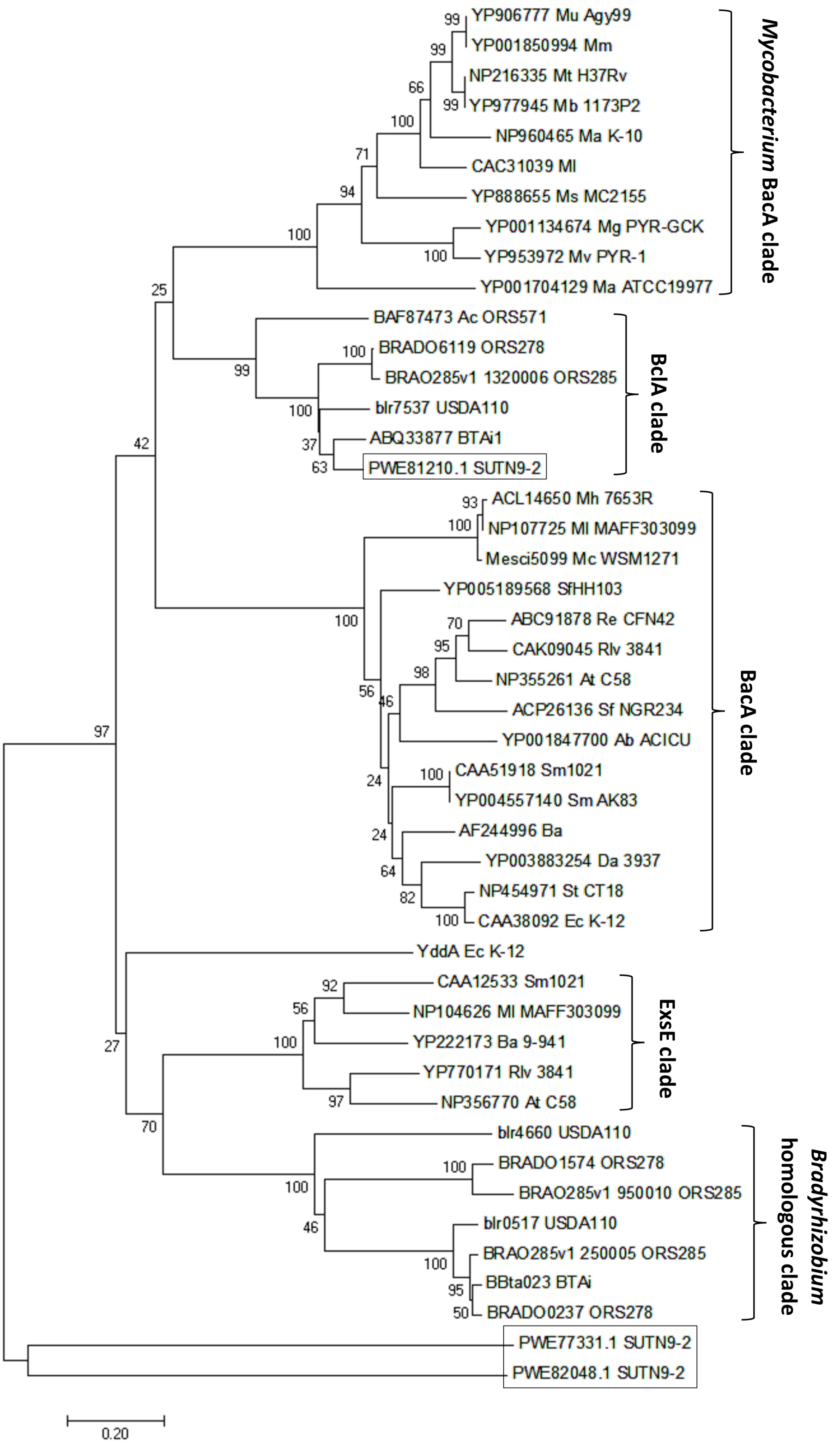

Fig. S6. Phylogenetic tree base on SbmA_BacA domain proteins. The neighbor-joining tree was generated from a ClustalW alignment with bootstrap 500 replicates. Protein sequences used for the alignment and tree generation are provided and adapted from Guefrachi *et al*. (23).
